# A web-based survey of UK pharmacists to assess the effectiveness of Viagra Connect^®^ additional risk minimisation measures

**DOI:** 10.1007/s11096-021-01339-7

**Published:** 2022-04-05

**Authors:** Joanna Lem, Janine Collins, Terry Maguire, Rachel E. Sobel

**Affiliations:** 1 Safety Surveillance Research, Worldwide Research and Development, Pfizer Inc, New York, NY USA; 2 United BioSource LLC, European Risk Management, Geneva, Switzerland; 3 School of Pharmacy, Queen’s University Belfast, Belfast, UK; 4 Worldwide Development, Pfizer Inc , New York, NY USA

**Keywords:** Additional risk minimisation measures (aRMM), Behind-the-counter (BTC), Community pharmacists, Effectiveness evaluation, Pharmacy medicine, Sildenafil citrate, Survey

## Abstract

*Background* To support reclassification in the UK of sildenafil citrate (50 mg) from prescription-only medicine to a pharmacy medicine (P status) under the brand name “Viagra Connect^®^”, additional risk minimisation measures were implemented that included training materials and an optional checklist to assist community pharmacists in the safe supply of Viagra Connect^®^ to suitable patients. *Objective* To evaluate the effectiveness of Viagra Connect^®^ additional risk minimisation measures by assessing community pharmacists’ participation in training, their knowledge of key risk messages, and utilisation of the checklist. *Setting* A post-authorisation safety study implemented as a web-based survey, conducted in a representative population of UK community pharmacists. *Method* A random sample of community pharmacists who received at least 1 request to supply Viagra Connect^®^ within the past 6 months completed an online questionnaire of 33 closed-ended questions/statements with multiple-choice responses. Data were summarised using descriptive statistics. *Main outcome measure* Knowledge of key risk messages and dispensing practices communicated in the additional risk minimisation measures. *Results* The survey was completed by 345 community pharmacists. Respondents displayed a high level of knowledge of key risk messages, with ≥80 % selecting correct answers for 43/51 items. Nearly all respondents (90.1 %) reported that the training materials were useful/very useful, and reported using the checklist at the point of supply (91.9 %). Counselling of patients who requested Viagra Connect^®^ was generally considered a positive exercise. *Conclusions* The Viagra Connect^®^ additional risk minimisation measures were effective for education of community pharmacists and to ensure safe supply of Viagra Connect^®^ behind-the-counter to patients.

**Supplementary Information:**

The online version contains supplementary material available at 10.1007/s11096-021-01339-7.

## Impacts on practice

- Reclassification in the UK of sildenafil citrate (50 mg) from a prescription-only medicine to behind-the-counter availability in pharmacies (P status) under the brand name “Viagra Connect^®^” has increased the role of community pharmacists in facilitating patient counselling, determining patient suitability for use of Viagra Connect^®^, and for directing patients to their doctor when needed.
- This study demonstrated that community pharmacists have a good working knowledge of the Viagra Connect^®^ additional Risk Minimisation Measures, which included tailored educational materials and decision aids intended to mitigate risks around supply of Viagra Connect^®^ without a prescription.
- Community pharmacists widely utilise the *Viagra Connect*^®^ *Pharmacy Checklist* at the point of supply and are confident in counselling patients who request Viagra Connect^®^. When pharmacists are uncertain of whether to supply the medicine, they tend to take a risk-averse approach and refer patients back to their doctor.
- Assessing the effectiveness of additional risk minimisation measures in the behind-the-counter setting is uncommon and the methodology employed may be repurposed to future medications reclassified to pharmacy medicines.

## Introduction

Sildenafil citrate, a phosphodiesterase type-5 inhibitor (PDE5I), is first-line treatment for erectile dysfunction (ED) [1, 2] and is indicated for men ≥18 years of age [3]. In November 2017, the UK Medicines and Healthcare products Regulatory Agency (MHRA) approved reclassification of sildenafil citrate (50 mg) from a prescription-only medicine (POM) to a pharmacy (P) medicine, for dispensing under supervision of a pharmacist (Viagra Connect^®^) [4]. Subsequently, Ireland and Norway also permitted supply of Viagra Connect^®^ behind-the-counter (BTC); however, sildenafil citrate remains a POM in most countries.

In the EU and UK, safety concerns associated with medicinal products are typically addressed by routine risk minimisation measures (RMMs), such as product information or package inserts, to ensure benefits outweigh the risks [5]. In certain circumstances, routine RMMs may not be sufficient and additional RMMs (aRMMs) are needed to address specific safety concerns. aRMMs are used to guide appropriate patient selection, support on-treatment monitoring and/or management of adverse reactions, minimise risk of medication error, and/or ensure appropriate administration when this cannot be achieved through product information/labelling alone [6]. Pharmacists working in the community (community pharmacists) play an important role in facilitating discussions and counselling men seeking to purchase Viagra Connect^®^ [4]. Community pharmacists were therefore target recipients for the UK aRMMs programme for Viagra Connect^®^, which, in agreement with the MHRA, aimed to minimise the risk of Viagra Connect^®^ being supplied to patients unsuitable to take the product without consultation with their doctor [4]. The UK aRMMs for Viagra Connect^®^ included the *Essential Information for the Supply of Viagra Connect*^*®*^ training guide and standalone checklist, an optional tool to help pharmacists assess patient suitability (version 01/18/2018; Online Resource 1) [7]. The checklist gauges overall fitness for sex by determining if he gets out of breath or experiences chest pain during physical activity, and helps identify men with cardiovascular problems, or taking contraindicated medications that would preclude them from safely using Viagra Connect^®^. The checklist also instructs pharmacists to provide lifestyle advice, and advise patients to consult their doctor within 6 months for a clinical review of potential underlying conditions and risk factors associated with ED [4, 7]. A patient record tear-off slip includes follow-up advice and should be presented to a pharmacist when they next request Viagra Connect^®^.

The training document and checklist were developed following discussions with practising pharmacists, general practice doctors, and 2 urology specialists to identify training needs of pharmacists supplying Viagra Connect^®^. Drafts were reviewed at a workshop of practising pharmacists and agreed with the MHRA. Findings and recommendations were incorporated into updated training materials, which were offered by the marketing authorisation holder to community pharmacists in a variety of modalities to ensure the widest opportunity for learning, including online resources, regional meetings, printed materials, and face-to-face. Training materials were supplied from February 2018, before launch of Viagra Connect^®^ on March 27, 2018. These materials (now updated) continue to be an important resource for pharmacists. Some pharmacists may have had experience supplying PDE5Is as a consequence of patient-group direction (PGD) training programmes [8]. Under UK legislation, PGD permits express healthcare professionals to supply medications to pre-defined patients without prescription [9, 10].

### Aim

This post-authorisation safety study, an MHRA regulatory commitment, was a survey of pharmacists to evaluate the effectiveness of UK Viagra Connect^®^ aRMMs in the community pharmacy setting. Effectiveness was evaluated by assessing community pharmacists’ participation in the training, knowledge of key risk messages (KRMs), and utilisation of the optional checklist when dispensing.

### Ethics approval

The study was screened through the Medical Research Council and National Health Service Research Authority Research Ethics Committee algorithm (http://www.hra-decisiontools.org.uk/ethics/) and responses to this assessment determined that specific ethical approval was not required. Informed consent was obtained by respondents as part of the survey. The purpose of the survey, how the data would be reported, and participant confidentiality were explained in the survey invitation.

### Key risk messages

KRMs were communicated in the Viagra Connect^®^ training materials and optional checklist. Part 1 communicated KRMs when determining patient suitability for Viagra Connect^®^: (1) supply criteria, (2) cardiovascular health, (3) concomitant medications, and (4) enquiring about concomitant medical conditions (Table 1). Part 2 communicated KRMs to consider during consultation: (1) possible causes of ED, (2) advising patients to stop taking Viagra Connect^®^ and seek medical attention if they experience serious side effects, (3) advising patients to consult their doctor within 6 months of first purchase, and (4) advising patients who have *not* been supplied Viagra Connect^®^ by a pharmacist to consult their doctor (Table 1).

**Table 1:** Key risk messages assessed in the survey

*Part 1: KRMs for pharmacists to consider when determining the suitability of a patient for Viagra Connect*^*®*^
Supply criteria
Patient’s cardiovascular health should be considered by a pharmacist when dispensing Viagra Connect^®^
Patient’s concomitant medication use should be assessed by a pharmacist when dispensing Viagra Connect^®^
Pharmacists should ask patients about concomitant conditions
*Part 2: other KRMs for pharmacists to consider during consultation*
Pharmacists should consider possible causes of ED
Pharmacists should advise patients to stop taking Viagra Connect^®^ and seek medical attention immediately if they experience any serious side effects
Pharmacists should advise all men who seek to purchase Viagra Connect^®^ to consult their doctor within 6 months of their first behind-the-counter purchase
Pharmacists should advise men who have not been supplied the product to consult their doctor

*ED* erectile dysfunction, *KRMs* key risk messages

## Methods

### Study design

This cross-sectional survey was conducted between 28 January and 31 March 2019. Before implementing the full survey, a pilot was conducted among 42 pharmacists (July–August 2018) to ensure implementation was optimal, that questions performed as intended, and to gain insight into recruitment. The pilot confirmed that the invitation and respondent pool were representative of UK pharmacies and no changes were required to the protocol. The full survey was launched ~ 9 months after distribution of the aRMMs materials to allow time for pharmacists to complete the training, and gain experience counselling patients and dispensing Viagra Connect^®^. All aspects of the survey were anonymous. Effectiveness of the aRMMs was evaluated by assessing pharmacists’ knowledge of KRMs contained in the Viagra Connect^®^ training materials, participation in training, and utilisation of the optional checklist and tear-off slip when dispensing (Online Appendix).

### Survey population

Community pharmacists were recruited via the National Pharmacy Database, which contains > 14,000 pharmacy records and is representative of the UK pharmacy population. The sample was generated to be representative of the UK pharmacist population, comprising 49.2 % large multiple-pharmacy chains (≥ 100 outlets), 12.4 % small multiple-pharmacy chains (6–99 outlets), and 38.4 % independent pharmacies (1–5 outlets). Pharmacies were selected at random.

Practising community pharmacists who reported ≥ 1 face-to-face request for Viagra Connect^®^ in the past 6 months, and consented to participate, regardless of prior training, were eligible. Pharmacists who indicated that they or an immediate family member currently worked for a pharmaceutical company, contract research organisation, the marketing authorisation holder, European Medicines Agency, or MHRA were not eligible. Online only pharmacists who did not conduct face-to-face consultations were excluded.

### Survey sample

Data protection requirements were complied with before contacting pharmacists. A random sample of pharmacists across 4000 pharmacies were sent postal invitations including a weblink to the survey. The purpose of the survey, how data would be reported, and confidentiality were explained in the invitation. Email reminders were sent after 1 week and 1 month of initial invitation. Each pharmacy received a single-use code to exclude duplicate entries by multiple respondents. If > 1 pharmacist per pharmacy wanted to participate, an additional code was requested. Respondents were offered a single financial remuneration for a completed survey, based on fair-market-value of expected completion time.

### Online survey

Data were collected via a structured, self-administered online questionnaire. The questionnaire comprised 33 closed-ended questions with multiple-choice responses that covered the study objectives and screening, demographics, experience with Viagra Connect^®^, utilisation of the checklist and tear-off slip, attitudes towards patient counselling and towards Viagra Connect^®^ training.

### Statistical analysis

A sample of 200 completed surveys was planned, based on statistical and practical considerations. A completed survey was defined when all questions relevant to participant’s responses (following skip logic) were answered. Only data from completed surveys were included. Data were summarised using descriptive statistics. Frequency distributions with 95 % CIs were calculated using the Clopper–Pearson method. aRMMs were considered effective if ≥ 80 % of pharmacists provided correct answers to questions pertaining to KRMs. This level was agreed upon as a reasonable threshold, as there are no established criteria or published literature to provide valid thresholds for measuring effectiveness of aRMMs.

## Results

### Survey participants

From 4000 invited pharmacies, 387 were screened (response rate: 9.7 %), and 357 were eligible (eligibility: 92.2 %). In total, 345 eligible respondents completed the survey (completion rate: 96.6 %) (Online Resource 2). Mean completion time was ~19.6 min. Respondents tended to be male (69.9 %), 41–60 years of age (40.3 %), and had been dispensing medications for ≥ 11 years (63.2 %). (Table 2). Most pharmacists were located in an urban setting (73.9 %), and nearly half worked for a large pharmacy chain.

**Table 2:** Characteristics of pharmacist respondents and pharmacy settings

	Pharmacists (n = 345) n (%)
*Male*	241 (69.9)
*Age, y*	
22–30	68 (19.7)
31–40	117 (33.9)
41–60	139 (40.3)
> 60	21 (6.1)
*Number of years working as a pharmacist*	
< 2	24 (7.0)
2–5	39 (11.3)
6–10	64 (18.6)
11–20	99 (28.7)
> 20	119 (34.5)
*Pharmacy location*	
Urban	255 (73.9)
Rural	90 (26.1)
*Pharmacy setting*	
Large multiple-pharmacy chain (≥ 100 outlets)	157 (45.5)
Small multiple-pharmacy chain (6–99 outlets)	61 (17.7)
Independent pharmacy (1–5 outlets)	127 (36.8)
*Estimated number of patients that requested Viagra Connect*^*®*^* (past 6 months)*	
1–2	23 (6.7)
3–5	65 (18.8)
6–10	82 (23.8)
11–20	73 (21.2)
21–30	44 (12.8)
≥ 31	58 (16.8)

#### Pharmacists’ knowledge of KRMs–Part 1

Five of 28 items assessed knowledge of KRMs in Part 1. At least 80 % of pharmacists selected correct responses to 24/28 items (Table 3). The majority correctly responded to questions concerning concomitant diseases which may be contributing to ED. The lowest correct response rate was observed for “*Men who had a heart attack or stroke > 6-months ago should not be supplied Viagra Connect*^*®*^ *but should be referred to their doctor*”, with 41.4 % correctly answering “False”. At least 80 % of respondents correctly answered questions concerning concomitant medications not recommended with Viagra Connect^®^ (Table 3). Questions regarding suitability of patients to use Viagra Connect^®^ when on a different dose of sildenafil or other ED treatment(s), when taking riociguat for lung problems, or beta-blockers (correct answers: “false”, “no”, “yes”, respectively) did not reach ≥ 80 % correct response rate.

**Table 3:** Responses to all questions in Part 1 (knowledge of key risk messages when determining patient suitability for Viagra Connect^®^)

Survey question	Correct answer	Pharmacists (n = 345) n (%)	95 % CI^a^
*Items with < 80 % correct responses*			
Please answer True/False/Don’t Know for the following statements:			
Men using a different dose of sildenafil or another ED treatment can also have Viagra Connect^®^	False	262 (75.9)	71.1–80.4
The following patients should NOT be supplied with Viagra Connect^®^ but should be referred to their doctor. Complete True/False/Don’t Know			
Men who had a heart attack or stroke > 6 months	False	143 (41.4)	36.2–46.8
Look at the list of drugs below and select whether or not they can be used with Viagra Connect^®^. Complete Yes/No/Don’t Know			
Riociguat for lung problems	No	248 (71.9)	66.8–76.6
Beta-blockers	Yes	264 (76.5)	71.7–80.9
*Items with ≥ 80 % correct responses*			
Please answer True/False/Don’t know for the following statements:			
Viagra Connect^®^ is only intended for use by men over 45 years of age who are experiencing ED	False	319 (92.5)	89.2–95.0
Viagra Connect^®^ must not be supplied to men who do not have ED	True	316 (91.6)	88.2–94.3
Men currently prescribed 50 mg sildenafil can be supplied Viagra Connect^®^ if (a) they meet the criteria for pharmacy supply, and (b) they do not take more than 50 mg daily	True	309 (89.6)	85.8–92.6
*The following patients should NOT be supplied with Viagra Connect*^®^ *but should be referred to their doctor. Complete True/False/Don’t Know*			
Those who have been advised by their doctor that they are not fit enough for physical or sexual activity	True	341 (98.8)	97.1–99.7
Those who experience breathlessness or chest pain with light or moderate physical activity (e.g. walking briskly for 20 min or climbing 2 flights of stairs)	True	340 (98.6)	96.7–99.5
* A patient with which of the following cardiovascular conditions MAY be supplied with Viagra Connect?*^*®*^ Complete Yes/No/Don’t Know			
Low blood pressure	No	276 (80.0)	75.4–84.1
Uncontrolled hypertension	No	330 (95.7)	92.9–97.5
Controlled hypertension	Yes	296 (85.8)	81.7–89.3
Unstable angina	No	333 (96.5)	94.0–98.2
Arrhythmia	No	283 (82.0)	77.6–85.9
Valvular heart disease	No	284 (82.3)	77.9–86.2
Cardiomyopathy	No	298 (86.4)	82.3–89.8
Left ventricular outflow obstruction or aortic narrowing	No	292 (84.6)	80.4–88.3
Severe cardiac failure	No	338 (98.0)	95.9–99.2
*Look at the list of drugs below and select whether or not they can be used with Viagra Connect*^*®*^.* Complete Yes/No/Don’t Know*			
Nitrates for chest pain	No	333 (96.5)	94.0–98.2
Ritonavir for HIV infection	No	290 (84.1)	79.8–87.8
Omeprazole or other PPIs	Yes	321 (93.0)	89.8–95.5
CYP3A4 inhibitors (e.g. saquinavir, cimetidine, itraconazole, ketoconazole, erythromycin or rifampicin, diltiazem)	No	289 (83.8)	79.4–87.5
Alpha-blockers (e.g. alfuzosin, doxazosin, tamsulosin)	No	290 (84.1)	79.8–87.8
Recreational drugs called ‘poppers’ (e.g. amyl nitrite)	No	329 (95.4)	92.6–97.3
*Patients with the following concomitant diseases should NOT be supplied with Viagra Connect*^*®*^ *but should be referred to their doctor. Complete True/False/Don’t Know *			
Hepatic disease	True	284 (82.3)	77.9–86.2
Severe renal impairment	True	291 (84.3)	80.1–88.0
Osteoarthritis or other musculoskeletal disorders	False	310 (89.9)	86.2–92.8
Loss of vision due to optic nerve damage or inherited eye disease (such as retinitis pigmentosa)	True	296 (85.8)	81.7–89.3

*CI* confidence interval, *ED* erectile dysfunction, *PPI* proton pump inhibitor

^a^95 % exact two-sided CIs are calculated using the Clopper–Pearson method

#### Pharmacists’ knowledge of KRMs–Part 2

Four questions consisting of 23 items were used to assess knowledge of KRMs in Part 2. At least 80 % of respondents correctly answered 19/23 items (Table 4). At least 80 % correctly identified that patients should be advised to stop taking Viagra Connect^®^ and seek immediate medical help if they experience chest pains, persistent/painful erections (> 4 h), loss of vision, or an allergic reaction. Below 80 % correctly identified that patients who experience headache or nausea do *not* need to stop taking Viagra Connect^®^ and seek medical attention (correct answers were “false”, Table 4). At least 80 % correctly identified conditions that may cause ED, and that patients with evidence of undiagnosed depression, anxiety, or excessive alcohol use should receive lifestyle advice and follow-up with their doctor. Below 80 % correctly identified hypertension and hypercholesterolemia as possible causes of ED (Table 4). Most respondents understood that all patients supplied Viagra Connect^®^ should be offered lifestyle advice, and advised to consult their doctor within 6 months of first supply, and that patients whose request for Viagra Connect^®^ was refused should be advised to contact their doctor.

**Table 4:** Responses to all questions in Part 2 (knowledge of key risk messages during consultation)

Survey question	Correct answer	Pharmacists (n = 345) n (%)	95 % CI^a^
*Items with < 80 % correct responses*			
*Which of the following MAY be a cause of ED and should be considered when assessing a patient for suitability for Viagra Connect?*^*®*^ *Please select True/False/Don’t Know*			
Hypertension	True	242 (70.1)	65.0–74.9
Hypercholesterolemia	True	196 (56.8)	51.4–62.1
*Complete True/False/Don’t know for the following statement: patients should be advised to STOP TAKING Viagra Connect*^*®*^ *and seek medical attention immediately if they experience the following symptoms:*			
Headache	False	263 (76.2)	71.4–80.6
Nausea	False	274 (79.4)	74.8–83.6
*Items with ≥ 80 % correct responses*			
*Which of the following MAY be a cause of ED and should be considered when assessing a patient for suitability for Viagra Connect?*^*®*^ * Please select True/False/Don’t Know*			
Undiagnosed depression	True	308 (89.3)	85.5–92.3
Anxiety	True	324 (93.9)	90.8–96.2
Excessive alcohol use	True	335 (97.1)	94.7–98.6
Diabetes mellitus	True	322 (93.3)	90.2–95.7
Cardiovascular disease	True	311 (90.1)	86.5–93.1
Osteoarthritis	False	290 (84.1)	79.8–87.8
*If a patient requests Viagra Connect*^*®*^ *but shows evidence of undiagnosed depression, anxiety, or excessive alcohol use, which action should you take? Please select True/False/Don’t Know*			
Provide lifestyle advice	True	336 (97.4)	95.1–98.8
Recommend follow-up with his doctor	True	340 (98.6)	96.7–99.5
Please select True/False/Don’t Know for the following statements:			
Pharmacists should provide lifestyle advice to all men with ED who request Viagra Connect^®^	True	299 (86.7)	82.6–90.1
Lifestyle advice includes losing weight, stopping smoking, cutting back on alcohol and recreational drugs, exercising regularly, and reducing stress	True	344 (99.7)	98.4–100.0
All patients supplied with Viagra Connect^®^ should be advised to consult their doctor within 6 months from the first supply	True	301 (87.2)	83.3–90.6
All patients who are refused Viagra Connect ^®^ should be advised to contact their doctor	True	312 (90.4)	86.8–93.3
*Complete True/False/Don’t Know for the following statement: patients should be advised to STOP TAKING Viagra Connect*^*®*^ *and seek medical attention immediately if they experience the following symptoms:*			
Chest pains	True	344 (99.7)	98.4–100.0
Persistent and sometimes painful erections lasting longer than 4 h	True	340 (98.6)	96.7–99.5
Dyspepsia	False	291 (84.3)	80.1–88.0
Sudden decrease or loss of vision	True	344 (99.7)	98.4–100.0
Allergic reaction (with symptoms of wheeziness, difficulty breathing, or dizziness)	True	345 (100.0)	98.9–100.0
Serious skin reactions (e.g. Stevens-Johnson syndrome)	True	340 (98.6)	96.7–99.5
Seizures or fits	True	339 (98.3)	96.3–99.4

*CI* confidence interval, *ED* erectile dysfunction

^a^95 % exact two-sided CIs are calculated using the Clopper–Pearson method

### **Awareness and utilisation of*Viagra Connect***^**®**^***Pharmacy Checklist***

Nearly all respondents were aware of the optional *Viagra Connect*^*®*^ *Pharmacy Checklist*, and the majority used this at point-of-supply (91.9 %; Table 5). Of the 28 (8.1 %) who did not/did not recall using the checklist, only 6 were unaware it existed. Nearly all respondents who utilised the checklist used it all the time (97.2 %). The majority (91.3 %) always provided the tear-off slip when supplying Viagra Connect^®^ (Table 5).

**Table 5:** Awareness and utilisation of the *Viagra Connect*^*®*^ *Pharmacy Checklist*

Survey question	Pharmacists (n = 345) n (%)
Do you use the optional *Viagra Connect* ^*®*^ *Pharmacy Checklist*at the point-of-supply?	
Yes	317 (91.9)
No	18 (5.2)
Don’t recall	10 (2.9)
From the list below, please select reason(s) for NOT utilising the optional *Viagra Connect*^*®*^ *Pharmacy Checklist*Select ALL that apply^a,b^	
I am familiar with the information, and I do not think I need the checklist to consult the patient	10 (35.7)
I use it only with new patients but not with returning patients	10 (35.7)
I used it for a while after the training but did not think I needed to continue to use it, as it was no longer required as a prompt	8 (28.6)
I was not aware of the checklist	6 (21.4)
I am PGD-trained (or equivalent) for PDE-5is	4 (14.3)
Other	1 (3.6)
N/A (answered “Yes” to use of *Checklist* at point-of-supply)	317
How frequently do you use the optional *Viagra Connect*^*®*^ *Pharmacy Checklist*?	
*For new requests for Viagra Connect*^*®*^^*a*^	
All the time for all customers presenting	308 (97.2)
Only used after first trained and no longer required as a prompt	8 (2.5)
Never	1 (0.3)
N/A (answered “No” or “Don’t recall” to use of *Checklist* at point-of-supply)	28
*For patients returning for repeat supplies of Viagra Connect*^®^^*a*^	
Only used after first trained and no longer required as a prompt	122 (38.5)
All the time for all customers presenting	111 (35.0)
Never	84 (26.5)
N/A (answered “No” or “Don’t recall” to use of *Checklist* at point-of-supply)	28
How frequently do you provide the patient with the tear-off slip contained on the bottom of the checklist?	
*If I have supplied Viagra Connect*^®^	
Always	315 (91.3)
Sometimes	23 (6.7)
Never	7 (2.0)
*If I have denied the patient’s request for Viagra Connect*^*®*^	
Always	219 (63.5)
Sometimes	59 (17.1)
Never	67 (19.4)
Before today, were you aware of the *Viagra Connect*^*®*^ *Pharmacy Checklist*?	
Yes	341 (98.8)
No	4 (1.2)

*PDE-5i* phosphodiesterase type-5 inhibitor, *PGD* patient-group direction

^a^Percentages calculated based on the sample presented with this question because of skip logic

^b^Percentages may not total 100 % as > 1 response could be selected

### **Participation in*Viagra Connect***^***®***^*training guide to pharmacists*

Of eligible respondents, 69.0 % (n=238) participated in the Viagra Connect^®^ training, with the most popular format being printed materials received in the mail, followed by online training supplied by their pharmacy (Online Resource 3). Of 107 respondents who did not participate, 47 could not recall if they had taken part. The most common reason given for not participating was lack of awareness (32.7 %), and 21 pharmacists (19.6 %) were already PGD-trained for PDE5Is. At least 80 % of respondents confirmed the *Essential Information for the Supply of Viagra Connect*^*®*^ training guide, *Viagra Connect*^*®*^ *Pharmacy Checklist*, and tear-off slip were extremely useful/very useful (90.1 %, 96.8 %, 82.9 %, respectively). Of 345 respondents, 161 (46.7 %) reported the training guide being their main reference source, and one-third referred primarily to the checklist for safe dispensing practices of Viagra Connect^®^ (Online Resource 4).

#### Pharmacists’ attitudes towards patient counselling

**Fig. 1:**
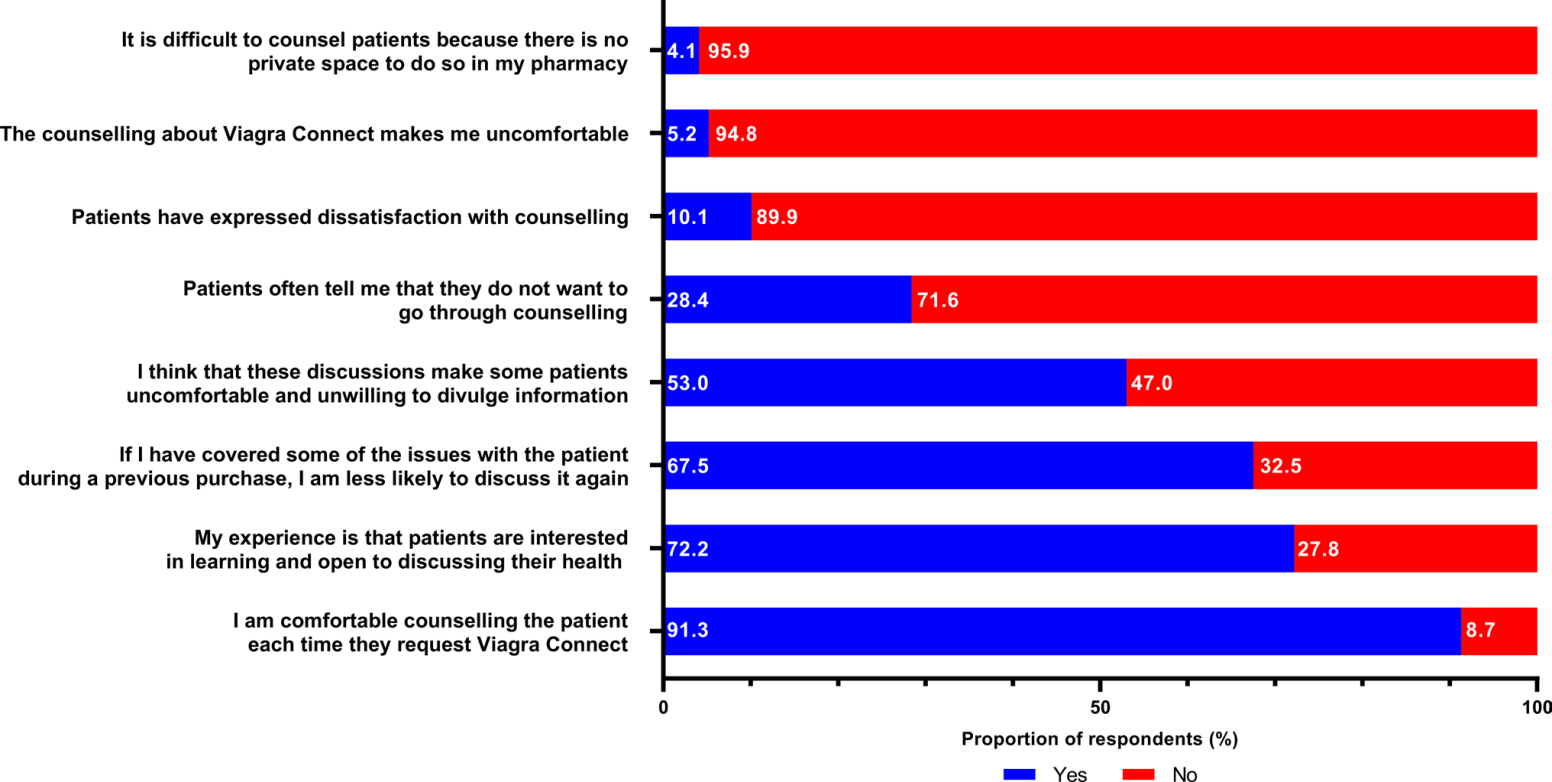
Pharmacists’ attitudes towards patient counselling

Of 345 respondents, the majority (91.3 %) were comfortable counselling patients. Most (72.2 %) agreed patients were interested in learning and open to discussing their health. However, 53.0 % reported that discussing Viagra Connect^®^ made some patients uncomfortable and unwilling to divulge information (Fig. 1). Only 5.2 % of respondents felt uncomfortable counselling about Viagra Connect^®^.

## Discussion

In recent years, there has been a move towards supporting self-care, and a key element of this has been the switching from POM to P-medicine, available BTC at pharmacies. We found that community pharmacists had a good level of knowledge and awareness of KRMs for Viagra Connect^®^, and, when unsure, acted in a risk-averse manner and did not supply Viagra Connect^®^. Awareness of the KRMs suggests the training materials were effective for educating community pharmacists on key aspects of its supply. To our knowledge, this was the first assessment of the effectiveness of aRMMs for a BTC-available product. Overall, a good level of uptake, engagement, and awareness of the Viagra Connect^®^ aRMMs programme was seen within the UK pharmacy setting. These results may be important for informing policy regulators in other countries considering reclassification of Viagra^®^ to non-prescription status.

With the availability of understandable information and online resources, society is better educated than ever about health and therapy options [11]. This has led to pharmacists experiencing increased responsibilities in facilitating self-care, with patients requesting medications for acute or long-term conditions [12]. While this study surveyed the main sources of pharmacists’ knowledge for dispensing Viagra Connect^®^, we acknowledge that pharmacists may have obtained information from other sources (Online Resource 4), and it is challenging to isolate the impact of a specific communication medium via a knowledge survey. Of the 31 % of respondents in this study who did not participate or recall participating in the Viagra Connect^®^ training, most cited a lack of awareness (32.7 %) or prior PGD-training for PDE5Is (19.6 %). It has been suggested that pharmacists lack confidence when supplying medications reclassified from POM to P-medicine [13], and see themselves as educators rather than decision-makers, deferring decision-making to other medical professionals in ambiguous cases [14]. Our survey suggests that when community pharmacists were unsure (i.e., < 80 % correctly answered 8/51 items), a risk-averse approach was adopted, and pharmacists directed patients to their doctor instead of supplying Viagra Connect^®^. These observations are consistent with the suggestion that pharmacists see safety as the over-riding concern with supply of over-the-counter or BTC medications [15]. Indeed, a study suggested that visits to physicians/nurse practitioners significantly increased amongst users following reclassification of Viagra Connect^®^ [16]. Our survey also demonstrated that the *Viagra Connect*^*®*^ *Pharmacy Checklist* was widely utilised (91.9 %), and nearly all pharmacists (96.8 %) found it useful/very useful. Although doctors and pharmacists play a critical role in encouraging self-care, they are reliant on information from consumer health sources [17]. Utilisation of the checklist at point-of-supply may help reduce ambiguity, provide support for gaps in knowledge, and embolden community pharmacists in their decision to supply Viagra Connect^®^. As a result of this survey, training materials were updated to improve knowledge of underlying health conditions as possible contributors to ED, and medications contraindicated with Viagra Connect^®^. These educational materials are still available to pharmacists [7].

Patient counselling is integral to a community pharmacist’s decision to dispense Viagra Connect^®^. We found pharmacists were comfortable counselling patients, and the majority perceived patients open to discussing their health, whereas previous studies suggested men were embarrassed to talk about their health and hesitant to seek treatment [18]. Amongst men diagnosed with ED, few pursue treatment [19]. In our study, 90 % of respondents reported patient satisfaction with counselling, suggesting patients were receptive to advice on ED and its treatment from pharmacists. With patients receptive to information from providers other than their primary care physician, and the good level of knowledge demonstrated by community pharmacists in counselling patients, there is real opportunity for patients to receive education and long-term management for ED independently of their doctor.

Our study should be considered in light of additional considerations. The overall response rate was 9.7 %, of whom 8.6 % completed the voluntary survey, despite email reminders and financial remuneration. However, the proportion of respondents was double that anticipated (~5 %). Low response rates are common among surveys of healthcare professionals [20–28], reported < 3 % in some publications [24, 25]. The present survey took ~19.6 min to complete, and although longer surveys (> 10 min) are associated with a lower response rate [29–31], our completion rate was high (96.6 %). We sought to recruit 200 pharmacists, but achieved 387 responses, allowing broader understanding of the implementation of the Viagra Connect^®^ training materials and checklist. To avoid limiting to pharmacists with experience supplying Viagra Connect^®^, those with > 1 patient request for Viagra Connect^®^ could participate. Despite our best efforts to ensure pharmacies surveyed were representative of the UK pharmacy population [32, 33], only 30 % of responding pharmacists were female, although women form the majority of practising pharmacists (62.0 %) [21]. This may be a consequence of the male-specificity of Viagra Connect^®^, although this was not specifically questioned. We cannot exclude the possibility that respondent characteristics, knowledge of the safety profile of sildenafil citrate, motivations, and general awareness of aRMMs differ between pharmacists who chose not to respond to the invitation. Furthermore, we do not know if pharmacists chose to complete the survey because they were better informed about ED or had an interest in pharmacovigilance, which may be an unintentional source of selection bias. Possible social desirability bias of respondents may also lead to an overrepresentation of positive responses to survey questions, although healthcare professionals may be less susceptible to this bias [34]. Finally, due to the subjective nature, it is not possible to accurately determine pharmacists’ attitudes towards practice and compliance. Further studies would be needed to quantify dispensing behaviours in pharmacies.

## Conclusions

Community pharmacists working across the UK had good working knowledge of the aRMMs for Viagra Connect^®^ (sildenafil citrate, 50 mg), including KRMs intended to minimise risk in patients requesting Viagra Connect^®^ without consultation with a physician. Utilisation and satisfaction with the *Viagra Connect*^*®*^ *Training Guide to Pharmacists* and optional *Viagra Connect*^*®*^ *Pharmacy Checklist* were high. Most community pharmacists indicated that the aRMMs materials were their main source of knowledge. Furthermore, information communicated in the aRMMs was well received and deemed effective for facilitating supply of Viagra Connect^®^ by community pharmacists. Overall, evaluating the effectiveness of aRMMs for a BTC product is uncommon. The methodology employed in our study may aid policy regulators in other countries considering future reclassification of Viagra^®^ to non-prescription status.

## Electronic Supplementary Material

Below is the link to the electronic supplementary material.

Supplementary Material 1
