# Supplementary material for: A web-based survey of UK pharmacists to assess the effectiveness of Viagra Connect® additional risk minimisation measures
Source: Int J Clin Pharm. 2022 Apr 5;44(3):608–18. doi: 10.1007/s11096-021-01339-7 (PMC9200697; doi:10.1007/s11096-021-01339-7)
Supplement: Supplementary file 1 — Supplementary Material 1 [file 11096_2021_1339_MOESM1_ESM.pdf]

Supplementary materials

A web-based survey of UK pharmacists to assess the effectiveness of the Viagra Connect® additional risk minimisation measures programme

Joanna Lem[1]* • Janine Collins[2] • Terry Maguire[3] • Rachel Sobel[4]

[1] Pfizer Inc, Safety Surveillance Research, Worldwide Research and Development, New York, NY, USA

[2] United BioSource LLC, European Risk Management, Geneva, Switzerland

[3] Queen's University Belfast, School of Pharmacy, Belfast, UK

[4] Pfizer Inc, Worldwide Development, New York, NY, USA (at the time the study was conducted)

***Corresponding author:** Dr Joanna Lem, JoannaAsia.Lem@pfizer.com

Online Resource 1

A copy of the *Essential Information for the Supply of Viagra Connect* training guide and the *Viagra Connect Pharmacy Checklist* available to pharmacists prior to the implementation of this survey.

Essential information for the supply of VIAGRA CONNECT®

50 mg film-coated tablets (sildenafil)

VIAGRA CONNECT®

CONTENTS

Introduction 5

SECTION 1
Understanding erectile dysfunction and VIAGRA CONNECT 7

SECTION 2
Who can use VIAGRA CONNECT? 13

SECTION 3
What is the VIAGRA CONNECT Checklist? 19

SECTION 4
Providing advice about VIAGRA CONNECT 23

SECTION 5
Patient supply scenarios 29

SECTION 6
Case study responses 35

SECTION 7
Essential information 37

References 41

INT ROD UCT ION

Introduction

This booklet is part of a package of information and training materials that Pfizer Consumer Healthcare have put in place to support the launch of VIAGRA CONNECT as an OTC treatment for Erectile Dysfunction. This document contains essential information you will need to know and consider when assessing men as suitable candidates for use of VIAGRA CONNECT.

While this booklet provides information on the key information to facilitate the assessment as to whether to supply VIAGRA CONNECT, an accompanying, comprehensive booklet, **"Supporting the pharmacist's role in the management of erectile dysfunction"**, provides information on erectile dysfunction, its causes, risk factors, co-morbid links, management, treatment options, VIAGRA CONNECT product information and pharmacist role in supporting the man.

This training can form part of your CPD training and once completed can be recorded at www.uptodate.org.uk

The sections in this booklet contain the following information:

SECTION 1

Understanding erectile dysfunction and VIAGRA CONNECT

Provides an understanding of VIAGRA CONNECT and how it works.

SECTION 2

Who can use VIAGRA CONNECT?

Details the types of men who can safely use VIAGRA CONNECT taking into account comorbidities and concomitant drug use.

SECTION 3

What is the VIAGRA CONNECT Checklist?

The VIAGRA CONNECT Checklist is provided as an optional tool to help accurately assess patients and to validate their suitability for treatment.

SECTION 4

Providing advice about VIAGRA CONNECT

Information on the advice you can offer patients who are suitable for VIAGRA CONNECT and follow-up advice for those who are found to be not suitable.

SECTION 5

Patient supply scenarios

Test your knowledge with a number of patient supply scenarios to which you can apply your professional judgement and check against the suggested supply outcomes.

SECTION 6

Case study responses

SECTION 7

Essential information

The essential information from the Summary of Product Characteristics.

1

Understanding erectile dysfunction and VIAGRA CONNECT®

What is Erectile Dysfunction?

Erectile Dysfunction (ED) has been defined as the inability to attain and/or maintain an erection hard enough for satisfactory sexual intercourse.[1] It can occur occasionally or frequently and men are able to self-recognise the condition, which can vary in severity from mild (a soft erection) to severe (no erection at all).

Causes of ED?

Understanding the causes and conditions associated with ED can help you manage the sufferer appropriately. In general, ED is due to 3 main causes:[2]

- Physical causes
- Psychological causes
- Some types of medication

An overview of the causes is detailed below; however, further in depth information is available in the accompanying training “Supporting the pharmacist’s role in the management of Erectile Dysfunction” which can be read in conjunction with this essential information guide.

1. Physical causes

ED usually has an underlying physical cause, and it is useful to bear this in mind when managing patients who have co-morbidities that may predispose them to ED. These could include hypertension, diabetes mellitus, hypercholesterolaemia or CV disease.[3]

2. Psychological causes:

Psychological causes inhibit normal stimuli to and from the brain and can interfere with the ability to achieve a normal erection. Although there can be a perception that ED is a psychological issue, the reality is, purely psychological causes account for only 1 in 10 cases.[4]

Psychological causes can include:[5]

- Performance anxiety in relation to ED and the fear that it will keep occurring
- Depression – the link with ED appears to be bidirectional. In depressed men, ED may be a symptom of depression, and in men with ED, the emotional stress commonly associated with loss of sexual function may lead to depression
- Antidepressant drugs, such as the tricyclics and selective serotonin reuptake inhibitors, can also cause ED[6]
- Lack of arousal and/or inhibition between partners
- Other sexual dysfunction (e.g. premature ejaculation)
- Major life stress such as; money worries, bereavement, etc

- Low self-esteem – this can be due to prior episodes of ED (thus a feeling of inadequacy) or can be the result of other issues unrelated to sexual performance
- Indifference – this may come about as a result of age and a subsequent loss of interest in sex, or stem from problems in a couple's relationship

3. Medication-induced

Although some drugs can directly cause erection problems (iatrogenic cause), many of the drugs shown in Table 1 are used to treat diseases that are themselves associated with ED.

Recreational drugs, including alcohol, can also cause erection problems.

Table 1: Medications that can cause ED[6–12]

Therapy area	Drug class or drug	Impact on ED
**Cardiovascular**	Diuretics	Not determined, but believed to interfere with smooth muscle relaxation
	ACE and ARB inhibitors	Interferes with smooth muscle relaxation
	Aldosterone antagonists	An anti-adrenergic action
	Beta-blockers	Affects sex hormones, impairs vasodilation of the corpora cavernosa
	Clonidine	Depresses adrenergic output
**Psychotropic**	Anti-depressants, e.g. selective serotonin reuptake inhibitors, tricyclics, monoamine oxidase inhibitors, lithium	Decreases arousal and desire
	Anti-psychotics, e.g. phenothiazines, butyrophenones	Increases prolactin levels
**Anti-epileptics**	Carbamazepine<br>Phenytoin<br>Barbiturates	Affects sex hormone levels

Table 1 continued

Therapy area	Drug class or drug	Impact on ED
**Endocrine drugs**	Testosterone antagonists/ oestrogen agonists<br>Anabolic steroids<br>Luteinising hormone-releasing hormone analogues	Affects androgen receptors, reducing sexual desire
**Recreational drugs**	Alcohol, heroin, cocaine, marijuana, methadone	Causes vasoconstriction and/or impacts on neurotransmitters in the erectile pathway
**Other**	H2 antagonists, ranitidine and cimetidine	Increases prolactin levels, reducing sexual desire
	Cytotoxics, e.g. cyclophosphamide, methotrexate	The effect of nausea and general malaise associated with such drugs often diminish libido

What is VIAGRA CONNECT and what is it used for?

VIAGRA CONNECT contains sildenafil 50 mg and is a pharmacy-only (P) medicine to treat erectile dysfunction (ED) in men aged 18 years and over. It is a well-established and well-tolerated treatment and provides men with access without prescription to a legal ED medication and professional health advice from the pharmacy.

As well as improving their erectile function, allowing men to fully engage in intercourse and/or masturbation, successful treatment with sildenafil can also have emotional benefits for many men with ED, helping them to regain their self-esteem, self-confidence and relationship satisfaction. It can improve their overall quality of life and thus positively impact the wider social implications of erection problems, such as depression.[13]

How does VIAGRA CONNECT work?

When a man is sexually stimulated, impulses from the brain reach the cavernous nerve which releases nitric oxide (NO) at nerve endings in the penis. The NO diffuses across the endothelial cells into smooth muscle and stimulates the enzyme, guanylate cyclase, to convert GTP into cyclic GMP (cGMP). Cyclic GMP starts a further cascade of biochemical reactions which results in smooth muscle relaxation and the cavernous bodies fill with blood and become rigid. Cyclic GMP is normally broken down by the enzyme PDE5 to GMP, which terminates the pathway and produces detumescence. VIAGRA CONNECT inhibits the action of this enzyme thus increasing cGMP levels and maintaining these levels for longer. Hence VIAGRA CONNECT helps a man attain and maintain an erection in response to sexual stimulation.[14]

In order for VIAGRA CONNECT to be effective, sexual stimulation is required.

Figure 1: VIAGRA CONNECT mode of action

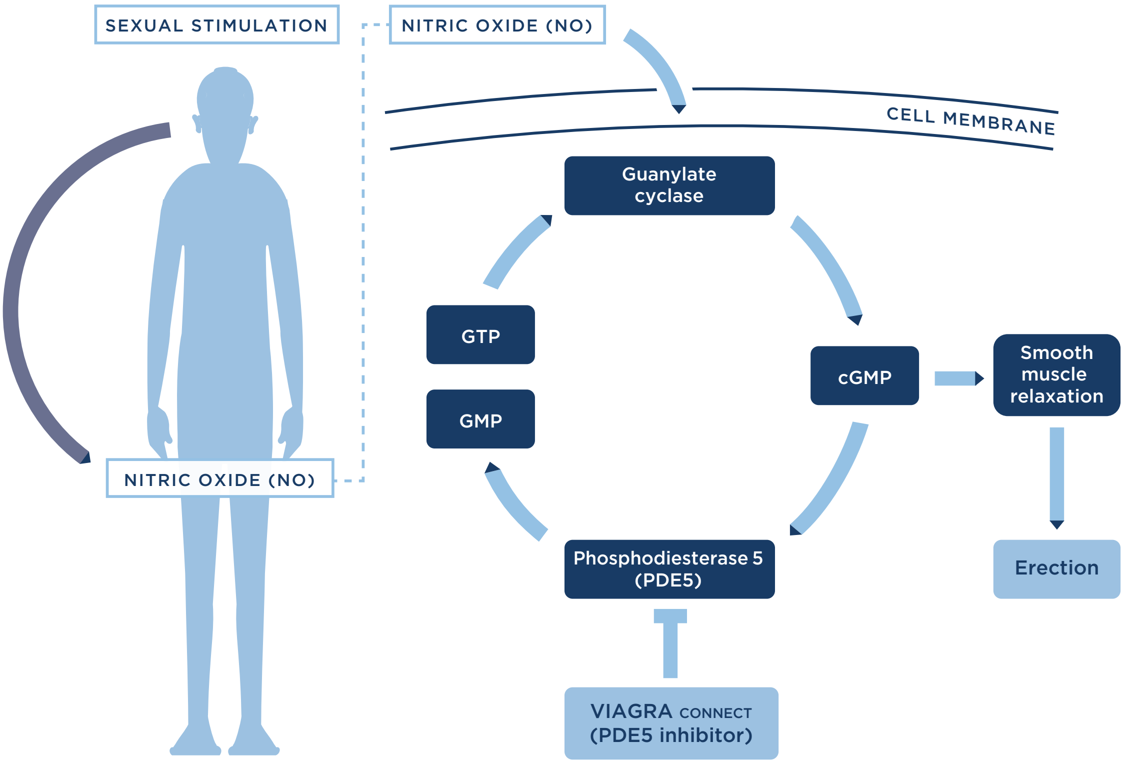

- PDE5 inhibitor augments the normal physiological response to sexual stimulation
- There is no effect in the absence of sexual stimulation

VIAGRA CONNECT Efficacy

VIAGRA CONNECT is an efficacious treatment for ED at the recommended 50 mg OTC dose. The efficacy of sildenafil has been demonstrated extensively in numerous clinical trials involving more than 23,000 men.

In some patients, 50 mg may not be efficacious and a referral to the GP is required as the dose may need to be increased to 100 mg. Patients who have renal or hepatic impairment, concomitant medication such as CYP3A4 inhibitors or alpha blockers or who have issues with tolerability should also be referred to their doctor as a 25 mg dose may be appropriate. The 25 mg and 100 mg doses are only available on prescription.

In fixed dose studies, the proportions of patients reporting that treatment improved their erections were 74% (50 mg).[15] These data show VIAGRA CONNECT is effective in men with ED and with a wide range of concomitant diseases.

2

Who can use VIAGRA CONNECT®?

Only adult men aged 18 years or older who have erectile dysfunction (ED) can use the product.

The active ingredient in VIAGRA CONNECT, sildenafil, is a part of the class of medicines known as Phosphodiesterase 5 Inhibitors (PDE5i) that are recommended as a first line treatment for ED by:

1. NICE's Clinical Knowledge Summaries[2]
2. The British Association for Urological Surgeons[4]
3. The British Society for Sexual Medicine[16]

VIAGRA CONNECT is licenced for packs of 2, 4 and 8 tablets (not all sizes may be marketed).

There are a number of contraindications or special warnings which mean that in certain circumstances the product cannot be used by men with ED, see below.

Who must not use VIAGRA CONNECT?

- **Men aged under 18 years**, as it not indicated for this age group. These men should be directed to their GP for follow up if they believe they have an issue relating to erectile function
- **Women**, as it is only indicated for men aged 18 years and over. For a woman interested in the product for their male partner, it is important to ask them to encourage their partner to visit the pharmacy or their doctor for additional advice
- **Men who do not have ED. It is important that you establish that the man has a problem getting or keeping an erection which is satisfactory for sexual performance.** VIAGRA CONNECT will not enhance men's erections or sexual performance and it will not help problems such as premature ejaculation. Men in the latter group should be directed to their GP for further advice
- **Men allergic to sildenafil** or any other ingredient in the medicine

Men with the following health problems must not use VIAGRA CONNECT:

- **Men with hypotension (<90/50 mm Hg) must not use VIAGRA CONNECT.** This is because the safety of sildenafil has not been studied in these sub-groups of patients, and its use is, therefore, contraindicated
- **Men with previously diagnosed mild, moderate or severe hepatic impairment** (e.g. liver cirrhosis), should be advised to talk with their GP about a suitable starting dose or alternative options for the treatment of ED. Men should be asked the questions included in the Checklist (see Section 3) relating to those diagnosed with liver disease or liver problems. If they are under the care of a doctor for liver problems, they will be aware of this and answer 'yes'. As such, this means that you must not supply the product and refer them to their GP
- **Men with previously diagnosed severe renal impairment.** In most cases, patients with severe renal impairment will have some signs or symptoms of the underlying issues and will be under the care of a GP or renal specialist. A question is included in the Checklist which asks the man whether he is suffering from severe kidney problems. If the answer to this is 'yes', then they should be advised to talk with their doctor about alternative options for the treatment of ED

- **VIAGRA CONNECT must not be used in patients with anatomical deformation of the penis** (such as angulation, cavernosal fibrosis or Peyronie's disease) **or in patients with sickle cell anaemia, multiple myeloma or leukaemia.** There is an increased risk of priapism with these patients and they should be directed to their GP for further advice
- **Sildenafil is contraindicated in patients who have loss of vision in 1 eye because of non-arteritic anterior ischaemic optic neuropathy (NAION)**, regardless of whether this episode was in connection with previous PDE5i exposure or not
- **Men who have an inherited eye disease such as retinitis pigmentosa** (a minority of these patients have genetic disorders of retinal phosphodiesterases). This is because the safety of sildenafil has not been studied in these sub-groups of patients, and its use is therefore contraindicated
- **Men who have any bleeding issues** (e.g. haemophilia) **or suffer from stomach ulcers** must not use VIAGRA CONNECT and should be directed to see their GP
- **Rare hereditary problems of galactose intolerance, Lapp lactase deficiency or glucose-galactose malabsorption** must not take VIAGRA CONNECT because of the lactose contained within the tablet
- **Men who have cardiovascular issues**, see section below

Men with the following cardiovascular (CV) issues must not use VIAGRA CONNECT:

- **Men who get very breathless or experience chest pains with light or moderate physical activity,** such as walking briskly for 20 minutes or climbing 2 flights of stairs should be referred to their GP[17]
- **Men who have been advised against sexual activity because of a CV problem.** This group of men are potentially at higher risk due to the burden of over activity on their heart and as such should be referred back to their GP
- **Men with recent history (in the last 6 months) of stroke or myocardial infarction** are also contraindicated from using VIAGRA CONNECT and will need to follow up with their GP
- **Patients with increased susceptibility to vasodilators include those with left ventricular outflow obstruction (e.g. aortic stenosis), or those with the rare syndrome of multiple system atrophy manifesting as severely impaired autonomic control of blood pressure.** Men with these conditions must not use the product without consulting a doctor
- **Patients previously diagnosed with the following must be advised to consult with their GP before resuming sexual activity:**
    - **uncontrolled hypertension**
    - **hypotension**
    - **unstable angina**
    - **moderate to severe valvular disease**
    - **left ventricular dysfunction**
    - **hypertrophic obstructive and other cardiomyopathies**
    - **significant arrhythmias**
    - **severe cardiac failure**

Note: Some low risk cardiovascular patients may be suitable for VIAGRA CONNECT, as long as their doctor has advised they can resume sexual activity: those with **asymptomatic controlled hypertension, mild valvular disease or who have had successful coronary artery bypass grafting, stenting or angioplasty.**

Men taking certain other medicines must not use VIAGRA CONNECT

- **Men taking nitrates (e.g. isosorbide mononitrate/dinitrate and glyceryl trinitrate), nitric oxide donors (e.g.nicorandil) or 'poppers' (e.g. amyl nitrate).** Consistent with its known effects on the NO cGMP pathway, sildenafil has been shown to potentiate the hypotensive effects of nitrates, and it is contraindicated for use with nitric oxide donors, nitrates, amyl nitrite (known as the recreational drug 'poppers'), sodium nitroprusside and nicorandil. These men should be directed to their GP for further advice
- **The co-administration of sildenafil with guanylate cyclase stimulators, such as riociguat, is contraindicated** as it may potentially lead to symptomatic hypotension. Again, these men will need to talk with a doctor for further advice
- **Men taking ritonavir**, a potent CYP3A4 inhibitor used in the treatment of HIV, are contraindicated due to the potential for increased blood levels of sildenafil in these patients. They should consult with their doctor for further guidance
- **Men already being treated with another PDE5i** or a higher or lower dose of sildenafil should not use VIAGRA CONNECT. Men taking 50 mg of sildenafil can use the product providing they meet the criteria for pharmacy supply and do not exceed 50 mg as a daily dose
- **Men taking CYP3A4 inhibitors or alpha-blockers** (see Table 2 for examples). These patients should be advised to speak with their GP about a lower suitable starting dose which is available on prescription

Table 2: Examples of CYP3A4 inhibitors and alpha-blockers

Antibiotics	Erythromycin, clarithromycin, rifampicin
Antifungals	Itraconazole, ketoconazole
Calcium channel blockers	Diltiazem, verapamil
H2-antagonists	Cimetidine
HIV-protease inhibitors	Amprenavir, fosamprenavir, atazanavir, darunavir, indinavir, lopinavir, ritonavir, saquinavir, tipranavir
Alpha blockers	Phenoxybenzamine, phentolamine, tolazoline, trazodone, alfuzosin, doxazosin, tamsulosin, prazosin, terazosin

Case studies

Case study 1:

How would you respond to the following patient request?

Mr. P is a man in his mid-50s who is new to your pharmacy. He has walked briskly to the pharmacy as it is raining and asks to see you. The first thing he states is: “I get out of breath so quickly these days; I could do with a sit down.” He then goes on to tell you he would like to purchase VIAGRA CONNECT.

How would you handle this conversation? Would you sell VIAGRA CONNECT in this situation?

See page 35 for case study responses

Case study 2:

How would you respond to the following patient request?

Mr. G is a 72-year-old man who has asked to talk with you about the 'new VIAGRA'. After questioning, you discover he is a smoker with a history of cardiovascular issues including angina, despite undergoing bypass surgery 3 years ago. He is on a range of cardiac medications including glyceryl trinitrate spray for his chest pains. He also has a number of other issues including type 2 diabetes and benign prostatic hypertrophy treated with tamsulosin. His wife has seen a newspaper advert for the product and reckons it is just what he needs.

How would you handle this conversation? Would you make a sale of VIAGRA CONNECT in this situation?

See page 35 for case study responses

3

What is the VIAGRA CONNECT® Checklist?

The VIAGRA CONNECT Checklist, on pages 20–21 of this document, has been provided as an optional tool to help you when assessing men to accurately validate their suitability for treatment. It also provides information which will enable you to offer valuable health advice for men regardless of whether or not they are suitable for the product.

There is no mandatory requirement to use the Checklist; it is your professional judgement to decide when and how to use as an aid in deciding whether to supply VIAGRA CONNECT to the man. The recommendation, however, is to use the supplied questions as a framework to assess suitability. You may also wish to check the patient's Summary Care Record or Patient Medication Record to check their concomitant conditions, such as hepatic disease or renal impairment, and medication intake, such as nitrate use.

The VIAGRA CONNECT Checklist includes an assessment of the man's overall fitness for sex, by determining if he gets out of breath or experiences chest pain when he undertakes physical activity. It is also important to understand if the man has any other medical issues that could mean the product is not suitable for him. These may include cardiovascular problems, such as a recent heart attack or stroke which would be revealed through questioning, so the following should be considered:

- There is a degree of cardiac risk associated with sexual activity, although the risk is small in those with stable CVD
- Checking the man's fitness for sex will help identify his CV risk; those with a low risk can be initiated on VIAGRA CONNECT and reminded to follow up with their GP within 6 months
- It also helps identify men at higher risk of CV problems who would benefit from further investigation by their doctor, ideally as soon as possible

Those patients who do not receive the product should be provided with the reason for non-supply and advised to go to the doctor. A tear off piece is included at the bottom of the Checklist for this purpose and contains a section to provide a written explanation of the reason for non-supply. The patient can then take the slip with them to the doctor to help start the conversation.

VIAGRA® CONNECT Pharmacy Checklist

50 mg film-coated tablets (sildenafil)

The following has been created as a useful aide-memoire to help determine whether your patient is suitable for Viagra Connect, or whether he should be seen by a doctor for further advice. Use of the checklist is optional, and you should use your professional judgement to decide when and how to use it. You may also wish to review the patient's Summary Care Record or Patient Medication Record to check concomitant conditions or medication use. The Essential Information for the Supply of Viagra Connect provides additional background information in relation to the supply of this product.

If the patient has previously been supplied with Viagra Connect, he should be asked if anything has changed with respect to his health status or medicines usage. There is no need to repeat the questions below prior to resupply in that case. Remind him to follow up with his doctor within the first 6 months of use. If any factors have changed, Sections 2–4 should be reviewed again. The repeat supply tear-off slip (below) can also be given to the man.

1. Who is Viagra Connect for?

Viagra Connect is only intended for men 18 years and older who are experiencing erectile dysfunction (ED) (i.e. difficulty in getting and/or maintaining an erection satisfactory for sexual performance). This product must not be supplied to men who do not have ED.

It is important to confirm if the man is already receiving treatment for the condition. Men currently prescribed 50 mg of sildenafil can be supplied this product if they meet the criteria for pharmacy supply, provided they do not take more than 50 mg daily. If the man is using a different dose of sildenafil or another ED treatment, he should be referred to his doctor.

Additional advice

You should consider possible causes of erectile dysfunction, such as undiagnosed depression, anxiety, excessive alcohol use and taking certain medicines. Examples of classes of medicines that cause ED include diuretics, anti-hypertensives, corticosteroids, anticonvulsants and recreational drugs. Whilst it may be appropriate to supply the product, you should provide lifestyle advice and recommend a follow-up with a doctor.

2. Check patient's cardiovascular (CV) health

If the patient answers **YES** to any of the following: **do not supply the product** and refer to the doctor. If you have any reason to consider, based on physical status, the patient should not be using this product, refer to the doctor.

- Y N Has your doctor advised that you are not fit enough for any physical and/or sexual activity?
- Y N Do you feel very breathless or experience chest pain with light or moderate physical activity, such as walking briskly for 20 minutes or climbing two flights of stairs?
- Y N Have you had a heart attack or stroke within the last 6 months?
- Y N Do you have any other heart problems or are you under a doctor's care for any of the following:
  - Low blood pressure or uncontrolled high blood pressure
  - Unstable angina (chest pain), irregular heart beat or palpitations (arrhythmia)
  - A problem with one of the valves in your heart (valvular heart disease)
  - A problem where the heart muscle becomes inflamed and does not work as well as it should (cardiomyopathy)
  - Heart problems causing blood flow issues (e.g. left ventricular outflow obstruction, aortic narrowing) or severe cardiac failure

3. Check concomitant medication use

Please check what other medicines the patient is taking.
If the patient answers **YES** to any of the following: **do not supply the product** and refer to the doctor.

- Y N Are you taking nitrates (nicorandil or other nitric oxide donors e.g. glyceryl trinitrate, isosorbide mononitrate or isosorbide dinitrate) for chest pain?
- Y N Are you using drugs called 'poppers' for recreational purposes (e.g. amyl nitrite)?
- Y N Are you taking riociguat or other guanylate cyclase stimulators for lung problems?
- Y N Are you taking ritonavir (for HIV infection)?
- Y N Are you taking any CYP3A4 inhibitors, e.g. saquinavir (to treat HIV infection), cimetidine (a heartburn treatment), itraconazole or ketoconazole (to treat fungal infections), erythromycin or rifampicin (antibiotics) or diltiazem (for high blood pressure)?
- Y N Are you taking any alpha-blockers, such as alfuzosin, doxazosin or tamsulosin, which are medicines to treat urinary problems due to enlarged prostate (benign prostatic hyperplasia) or occasionally to treat high blood pressure?

4. Check concomitant conditions

If the patient answers **YES** to any of the following: **do not supply the product** and refer to the doctor.

- Y N Do you have Peyronie's disease or any other deformation of the penis?
- Y N Have ever had loss of vision because of damage to the optic nerve (such as non-arteritic anterior ischaemic optic neuropathy [NAION]) or have an inherited eye disease (such as retinitis pigmentosa)?
- Y N Do you have galactose intolerance, Lapp lactase deficiency or glucose-galactose malabsorption?
- Y N Do you have previously diagnosed hepatic (liver) disease (including cirrhosis of the liver) or severe renal (kidney) impairment?
- Y N Do you have any of the following: sickle cell anaemia, multiple myeloma or leukaemia?
- Y N Do you have any bleeding issues (e.g. haemophilia) or have active stomach ulcers?

Dear Doctor,

Please can you review this patient in relation to erectile dysfunction. We had a discussion in my pharmacy, but he was not suitable for supply of Viagra Connect due to his [delete as appropriate] cardiovascular health/interacting medicines/other condition:

PHARMACY STAMP

....

Pharmacist signature:.. Date:..........................

Please keep this slip and present to the Pharmacist when you next wish to purchase Viagra Connect.

Before resupply your Pharmacist will need to assess you to ensure that there have been no changes in your health or medications since the last time you received the product.

PHARMACY STAMP

Pharmacist signature:.. Date:..........................

Points for counselling and other information

Please follow these points to ensure that your patient is counselled appropriately whether they are supplied the product or not.

Advice for men who have **not** been supplied Viagra Connect

Men who have not been supplied Viagra Connect because of their cardiovascular health, interacting medicines or another concern must be told to see their GP as soon as they can within 6 months. The tear-off slip (below) can be filled in and given to the man to facilitate his discussion with the doctor.

Advice for men who have been supplied Viagra Connect

Men should be advised:

- Viagra Connect is only intended for men 18 years and older who have erectile dysfunction (ED). Men who do not have ED will not benefit from using this product
- Take one tablet approximately 1 hour before planning to have sexual intercourse. Viagra Connect can start to work within 30 minutes
- Take with or without food, but Viagra Connect may take longer to work after a high-fat meal
- Do not take with grapefruit or grapefruit juice, as it may affect the way the medicine works
- The maximum recommended dosing frequency is one 50 mg tablet per day
- They may need to take Viagra Connect a number of times on different occasions (a maximum of one 50 mg tablet per day), before they can achieve a penile erection satisfactory for sexual activity. If, after several attempts on different dosing occasions, patients are still not able to achieve a penile erection sufficient for satisfactory sexual activity, they should be advised to consult a doctor
- Medicines containing any nitrates (e.g. glyceryl trinitrate, isosorbide mononitrate, isosorbide dinitrate, amyl nitrite also known as ‘poppers’), or nitric oxide donors (e.g. sodium nitroprusside or nicorandil), must NOT be used at the same time as Viagra Connect as this combination may lead to a dangerous fall in blood pressure
- Men should tell their doctor that they have started taking Viagra Connect, especially if they are started on any new medicines
- Remind patients about common side effects. These include: headache, flushing, dyspepsia, nasal congestion, dizziness, nausea, visual disturbance, cyanopsia (blue-tinted vision) and blurred vision

Note: If any of these become a concern, advise the patient to talk with a pharmacist or doctor.

Men should be advised to STOP TAKING Viagra Connect and seek medical attention IMMEDIATELY if they experience any of the following SERIOUS side effects. Side effects can be reported by patients or pharmacists via the Yellow Card Scheme at www.mhra.gov.uk/yellowcard.

- Chest pains: If this occurs before, during or after intercourse, they should get into a semi-sitting position and try to relax. Nitrates must NOT be used to treat chest pains
- A persistent and sometimes painful erection lasting longer than 4 hours
- A sudden decrease or loss of vision
- An allergic reaction. Symptoms include sudden wheeziness, difficulty breathing or dizziness, swelling of the eyelids, face, lips or throat
- Serious skin reactions such as Stevens-Johnson Syndrome (SJS) and Toxic Epidermal Syndrome (TEN). Symptoms may include severe peeling and swelling of the skin, blistering of the mouth, genitals and around the eyes, fever
- Seizures or fits

Follow up advice for all men

- ED can be associated with a number of contributing conditions, e.g. hypertension, diabetes mellitus, hypercholesterolaemia or cardiovascular disease. As a result, all men with ED should be advised to consult their doctor within 6 months for a clinical review of potential underlying conditions and risk factors associated with ED
- Provide appropriate advice on lifestyle factors and general healthy living, including:
  – Losing weight
  – Giving up smoking
  – Cutting back on alcohol/recreational drugs
  – Exercising regularly
  – Reducing stress
- You may also want to check if the man is buying products from unregulated sources. It is important to explain these products are not tested for their safety or effectiveness, may not contain the ingredients listed within them and are therefore potentially dangerous, unlike product sourced from a pharmacy and medicines obtained via prescription from the doctor

Your Pharmacist has supplied you with Viagra Connect today for your personal use.

Please ensure that you make an appointment to see your GP within 6 months of the date of first receiving Viagra Connect (see date on reverse).

You have not been provided with Viagra Connect for ED today as it may not be suitable for you to take without consulting your doctor. You should go to see your doctor to discuss other suitable options.

Please make an appointment to see your GP as soon as you can within the next 6 months and present this slip.

4

Advice for men who are suitable for VIAGRA CONNECT®

Advise patients:

- The product is only for men aged 18 years and over who have ED. Men who do not have ED will not benefit from this product
- To avoid taking nitrates (e.g. glyceryl trinitrate, isosorbide mononitrate, isosorbide dinitrate), nitric oxide donors (e.g. sodium nitroprusside or nicorandil) or amyl nitrite (“poppers”) as these can cause a dangerous drop in blood pressure when used in combination with VIAGRA CONNECT
- During any interaction with their doctor men should tell their doctor that they have started taking VIAGRA CONNECT, especially if they are started on any new medicines
- Men should not take after excessive drinking as VIAGRA CONNECT may be less effective. Excessive alcohol consumption can impact sexual function, as it acts as a central nervous system depressant and can impair motor and cognitive function
- How to take VIAGRA CONNECT:
    - Take 1 tablet approximately 1 hour before planning to have sexual intercourse or to masturbate
    - Swallow the tablet whole, with water
    - Do not take more than 1 tablet per day
    - VIAGRA CONNECT can start to work within 30 minutes, but men are still able to obtain an erection in response to sexual stimulation for up to 4 hours post-dose
    - VIAGRA CONNECT can be taken with or without food but may take longer to work after a high-fat meal
    - Avoid grapefruit juice as this may increase sildenafil levels in the blood
- For most men, VIAGRA CONNECT will work the first or second time they try it. For men who have not been able to have sexual intercourse for some time, it can take additional attempts to obtain maximum benefit. 74% of men will respond to VIAGRA CONNECT (sildenafil 50 mg).[15] If after several attempts (up to 8 separate dosing occasions) on different dosing occasions, patients are still not able to achieve a penile erection sufficient for satisfactory sexual activity, they should be advised to consult their doctor.
- One VIAGRA CONNECT tablet can be taken daily, men should be advised to visit their doctor within 6 months of starting the product for a general health check-up. Once reviewed by their doctor, they can continue using the product, providing there is no change in their circumstances (health or medication).
- If they experience any of the serious side effects detailed in the Side Effect section below, they should stop taking VIAGRA CONNECT and seek IMMEDIATE medical advice.

What if a patient takes more than the recommended dose of VIAGRA CONNECT?

Data show that at doses 16 times the VIAGRA CONNECT dosage, adverse reactions were similar to those seen at lower doses, but the incidence rates and severities were increased. If you are concerned a patient has taken an overdose, refer them immediately to the nearest Accident and Emergency facility or their on-call GP.

Are there any side effects associated with VIAGRA CONNECT?

Sildenafil is generally well tolerated and side effects reported in association with its use are usually transient and mild-to-moderate in intensity.

Patients who experience any of the following after taking VIAGRA CONNECT should be advised to STOP TAKING VIAGRA CONNECT and seek IMMEDIATE medical advice:

- **Chest pain, dizziness, feeling of faintness or nausea during or after sex. Nitrates must NOT be used to treat chest pains**
- **A persistent and sometimes painful erection that lasts more than 4 hours**
- **A sudden decrease in vision or hearing**
- **Allergic reaction including sudden wheeziness, difficulty breathing, dizziness or swelling of eyelids, face, lips and throat**
- **Serious skin reactions which include severe peeling and swelling of the skin, blistering of mouth, genitals and around the eyes, and fever**
- **Seizure or fits**

A full list of side effects experienced with VIAGRA CONNECT can be found in the SmPC.[15]

The most commonly reported side effects in the use of VIAGRA CONNECT are:

- Headache
- Facial flushing, hot flush
- Dyspepsia
- Nausea
- Nasal congestion
- Dizziness
- Visual disturbance, blurred vision and blue-tinted vision (cyanopsia)
- Temporary change in colour vision

Advice for men who are not suitable for VIAGRA CONNECT®

Please see Section 2 for men who must not take VIAGRA CONNECT.

If unsuitable for VIAGRA CONNECT, it is important to advise the man of the reason they are not suitable (e.g. it may be a contraindication or that he doesn't have ED). All men with ED should be referred to their GP to explore other suitable options and advised that they should do this within the next 6 months. Men should be encouraged to seek advice from their doctor for two reasons – because ED may be a symptom of other conditions that need medical attention and because, although VIAGRA CONNECT is not suitable for them, the doctor may be able to prescribe medicines which are suitable.

Advice for all men

ED can be associated with a number of underlying conditions, e.g. hypertension, diabetes mellitus, hypercholesterolaemia or cardiovascular disease. As a result, all men with ED should be advised to consult their doctor within 6 months for a clinical review of potential underlying conditions and risk factors associated with ED.

Lifestyle advice and guidance should be provided to all men, as it can help to reduce the risk factors for ED:

- Weight management
- Healthy eating
- Regular exercise
- Reducing stress
- Quitting smoking
- Moderating alcohol consumption
- Avoiding recreational drugs

As well as helping to improve ED, these changes can also improve general health and may help to reduce the risk of CVD.

For more detailed lifestyle advice please see the accompanying training "Supporting the pharmacist in the management of erectile dysfunction".

Case studies

Case study 3:

How would you respond to the following patient request?

Mr. W is back at your pharmacy having purchased VIAGRA CONNECT for the first time a few days ago. He tells you he's taken a tablet, but it didn't work and he doesn't know why.

How would you handle this conversation? What advice would you give?

See page 35 for case study responses

Case study 4:

How would you respond to the following patient request?

Mr. R is a 58-year-old smoker who, reluctantly, asks if there is anywhere he can speak to you in private. You take him into the consultation area, where you establish he has ED. Mr. R asks to buy 2 packs of VIAGRA CONNECT, but does not want to go into any details about his issue. You ask him how long he has had the problem and he explains that he has been drinking more since he lost his mother last year. He tells you his partner asked him to come to the pharmacy amid worries about his sexual and mental health.

How would you handle this conversation? Would you make a sale of VIAGRA CONNECT in this situation? What other advice could you offer?

Remember: ED may not be the only concern; Mr. R has some signs that could indicate untreated depression

See page 35 for case study responses

5

Patient supply scenarios

While the decision to supply VIAGRA CONNECT or not will be straightforward in the majority of cases, there will be some situations where the decision is less clear cut. In this section, you will find some less frequent, but more thought-provoking supply scenarios to consider.

Which of the following patients do you feel are suitable for VIAGRA CONNECT.

For some suggested actions see page 31.

S1

Mr. S is a 57-year-old man and has been a widower for 3 years. He has started a new relationship and is very anxious that this goes well and reveals that he has been having problems with his erectile function for the last 8 months. At his regular health check-up at his doctor 5 months ago, there was nothing untoward found in his assessment. He is in relatively good health and takes ibuprofen for osteoarthritis; he was a smoker (20-a-day) but gave up about 8 years ago.

S2

Mr. T has had difficulty controlling his hypertension over the last 12 months. After many changes (including following lifestyle advice), he has been stabilised for the past 3 months on a thiazide-related diuretic and an angiotensin-II receptor antagonist combination. Although he is happy with his cardiovascular status, he is having problems with his ability to keep an erection and wonders if VIAGRA CONNECT would be suitable for him.

S3

Mr. B has been discharged into the care of his GP having had a heart attack 9 months ago that resulted in bypass surgery. After an intense period of cardiac rehabilitation, he is no longer experiencing any chest pain or breathlessness. He explains he is interested in using VIAGRA CONNECT, as he and his wife have missed the physical side of their relationship and his GP has advised him to talk with you about the OTC options available. He is on a standard regimen of drugs including aspirin, a statin and an ACE inhibitor and he also takes a non-steroidal anti-inflammatory drug for osteoarthritis.

S4

Mr. F is a 36-year-old man and wants a quiet word with you. He is in good health and follows a good diet and exercise regime, but tells you his new girlfriend is hard to satisfy. While he has no issues with having sexual intercourse, he is finding it is taking him longer to recover post coitus and as such wants something to 'pep him up'.

S5

Mr. M came to see you a few weeks ago for a Medicines Use Review (MUR). During that process, you advised him to talk to his doctor about his prescription for a sublingual glyceryl trinitrate spray. Although he has held a prescription for this drug for a number of years, he has not needed to use this. His MUR shows he is on a standard combination of other drugs to control his symptoms, and as a result of your intervention, his doctor has removed the nitrate from his drug regimen. He is now back and is keen to discuss whether or not VIAGRA CONNECT would be suitable for him as he is experiencing erectile dysfunction. You see from his drug records there are no other contraindicated medicines and he is well with no overt cardiovascular related symptoms.

S6

Mr. A asks for VIAGRA CONNECT as he has recently found it more difficult to perform sexually. On questioning him, you note he is a diabetic and takes a number of tablets for the condition. He also tells you he likes a drink and has been using alcohol more frequently of late. The diabetic nurse has warned him that he may need to start using insulin as his sugar control is very poor.

S7

Mr. Y has been coming to your pharmacy on a regular basis and has been purchasing VIAGRA CONNECT. In fact, it is 7 months since his first purchase and although you have repeatedly reminded him he needs to follow up with his doctor, he hasn't done so. He has promised he will make an appointment right after he finishes in the pharmacy and tells you it has kept slipping his mind.

S8

Mr. K came into your pharmacy last week and after taking him through the VIAGRA CONNECT Checklist you supplied an 8-tablet pack of VIAGRA CONNECT. He is back this week and has asked for a further supply of 8 tablets.

S9

Mr. L is a regular patient who was started on a course of treatment with an SSRI for depression about 6 weeks ago by his GP. Whilst Mr. L is feeling much happier, he has noticed he has had trouble getting and keeping an erection. He is keen to try VIAGRA CONNECT.

S10

Mr. D is a 70-year-old gentleman who has recently started a new relationship. He tells you he is concerned about the quality of his erection and that he has difficulties maintaining this during sexual intercourse. You ask him about his medication history and he tells you he takes a product for BPH. On further questioning you ascertain the drug is finasteride, which he has been taking for 18 months. He has not had sex since he lost his wife 3 years ago, and he is keen to try VIAGRA CONNECT.

S11

Mr J is a 65-year-old man who is interested in purchasing VIAGRA CONNECT. You take him through the Checklist and when it comes to questions about his cardiovascular status, all you can ascertain from him is that he has some problems with his heart. As he is a regular customer you check his patient medication record and find he is prescribed the following in the last 12 months: Atenolol 100 mg, Hydrochlorothiazide 25 mg and 2.5 mg amiloride. What advice would you give him?

Patient scenarios: Suggested actions

S1

VIAGRA CONNECT can be supplied to Mr. S. From his history, the use of the Checklist which has helped ascertain his low CV risk and fitness for sex as well as your professional judgement, he would appear to be in good health. He has given up smoking and he regularly engages with his doctor. The information he has provided helps with the supply decision. For most men, VIAGRA CONNECT will work the first or second time they try it. For men who have not been able to have sexual intercourse for some time, such as Mr. S, it can take additional attempts to obtain maximum benefit (up to 8 separate dosing occasions). If he doesn't respond after 8 doses, he should be advised to consult with his GP. He should also inform his GP he is using the product when he next visits and this consultation should be within 6 months.

S2

VIAGRA CONNECT can be supplied to Mr. T as his hypertension is now controlled and he is not taking any contraindicated medicines. As an addition to using the Checklist and your professional judgement, it is clear that Mr. T is under the care of a doctor as he has been stabilised with respect to his hypertension. Given what Mr. T has told you it looks like he is fit for sex. Advise Mr. T of the likely side effects of VIAGRA CONNECT and get him to mention he is using the product to his doctor and that he should visit the doctor within 6 months. Advise him to come back if he has any problems or if there are any changes to his health or medication.

S3

VIAGRA CONNECT can be supplied to Mr. B as it has been more than 6 months since his heart attack and he is not taking any contraindicated medications. Provide Mr. B with the usual follow-up advice. The Checklist has meant that the CV health and fitness for sex has been evaluated and in this case the patient has shown very good signs of improvement in relation to his cardiovascular and general health. He has obviously followed his specialist's advice and has done well with his cardiac rehabilitation. It is important for Mr. B to mention he is using the product to his doctor and as usual he should be counselled on using the product correctly and to go back to the doctor within 6 months.

S4

VIAGRA CONNECT cannot be supplied to Mr. F as it does not help with shortening the refractory period in sexual intercourse. The drug is only indicated for men who have ED and have described their symptoms in line with the definition of the condition. The no supply slip should be completed and he should be advised to visit his doctor for further advice.

S5

VIAGRA CONNECT can be supplied to Mr. M. Following the questions on the Checklist, it has been ascertained that he is not taking any contraindicated medications and had no other overt CV issues. As the patient's GP is happy to remove his nitrate prescription and he seems in otherwise good health, he looks like a suitable candidate for the product. The Checklist should be used to provide advice to counsel Mr. M in using the product correctly and advise that he return to see his doctor within 6 months.

S6

VIAGRA CONNECT can be supplied to Mr. A. It is important that he is taken through the Checklist especially with a view on fitness for sex. It appears that he can be supplied the product, but in Mr. A's case there are other issues that warrant investigation, e.g. his increased alcohol use could be a symptom of depression, and he should be advised to speak with his GP and/or his diabetic nurse within 6 months.

S7

There are no restrictions within the SPC to prevent supply of the product, so the product can be supplied. However, the man should again be reminded that it is very important that he should see his GP and that this may be because there are underlying conditions which may be causing the ED and you may wish to elaborate on these further to help facilitate the man to see his GP. If you get to the point where you feel that the man has not taken your advice, it is longer than 6 months post supply and the man has continued not to see his GP, you are free to use your professional judgement and state you do not wish to supply until you are happy he has been assessed by his GP.

S8

Provided there has been no change since Mr. K completed the VIAGRA CONNECT Checklist, you can provide a further supply of VIAGRA CONNECT. Remind him the maximum dose is 1 tablet per day. It is also important to check that the drug is working for Mr. K and that he is deriving the benefit he needs. The message to see his GP within 6 months should be repeated.

S9

Provide Mr. L with positive encouragement that it is good to hear that the anti-depressant is working and that he is feeling happier and reiterate that despite this issue he should not stop taking the anti-depressants. State that ED can be a side effect of anti-depressants, but reinforce that VIAGRA CONNECT can be supplied. Make sure to advise Mr. L to tell his GP he has started taking VIAGRA CONNECT and that he has experienced ED. You should also counsel him on what he should do if VIAGRA CONNECT makes no difference and that in this circumstance he should not discontinue his SSRI medication but return to the GP immediately for further advice.

S10

VIAGRA CONNECT can be supplied to Mr. D as the Checklist has meant that you have ascertained he is fit for sex, he has good cardiovascular health and is not taking any contraindicated medicines. Provide advice on using the product, particularly as he has not had sex for 3 years, it may take several attempts before it works. For most men, VIAGRA CONNECT will work the first or second time they try it. For men who have not been able to have sexual intercourse for some time, it can take additional attempts to obtain maximum benefit (up to 8 separate dosing occasions). If it doesn't work after 8 different dosing occasions (a maximum of one 50 mg tablet per day) he should see his GP. He should also be advised to see the GP within 6 months of taking VIAGRA CONNECT for a health check.

S11

Mr J's medications could be causing erectile dysfunction as a side effect, and they are not contraindicated with VIAGRA CONNECT. His medications may also give an indication of the conditions he may have. He could be taking atenolol for hypertension, angina or cardiac arrhythmias, and the hydrochlorothiazide/amiloride combination for hypertension or congestive heart failure. Given he has had the same prescriptions for the past 12 months, it is likely that his blood pressure is stable, but you won't be able to know that all other conditions are controlled. It is important to check the patient's CV health status, including fitness for sex, as per the Checklist, as this may not be clear from his medications alone.

If following further evaluation using the Checklist, you decide that Mr J is suitable for VIAGRA CONNECT, it will be important to advise Mr J on how to use the product. If the CV health status and suitability cannot be deduced from your discussions with the man, then it is probably best to refuse supply and refer him to his doctor.

6

Case Study Responses

Case study 1

Given that ED can be an early sign of the development of underlying CVD and coupled with the fact Mr. P is getting out of breath quickly, it is important that he is advised to visit his doctor for a check-up. There is an opportunity to discuss Mr. P's lifestyle and provide him with some information around this, but it is important he is not supplied the product and advised to take further advice from his GP. This guide and the Checklist provide more information around these issues.

Case study 2

Mr. G falls in the category of patients using nitrates and in this case you must not supply VIAGRA CONNECT to him. Taking a nitrate and VIAGRA CONNECT together may lead to a significant drop in blood pressure. You should advise Mr. G of this fact and advise him to speak to his GP about this. In addition, it is important to provide counselling around his other medical issues and again ask him to speak to his doctor about his current health status. You can also offer him advice around his lifestyle choices.

Case study 3

For some patients VIAGRA CONNECT may not work on taking the first few doses. This can be for a variety of reasons; the man may have been out of practice for a while or may need a higher dose or an alternative treatment. With Mr. W, it is important to establish, how he used the product, e.g. did he take it on an empty stomach or after a meal? Did he take it one hour before he planned to have sexual intercourse?

Once correct use is established and advised on, it will be important to understand what his situation is. For example, is this a new relationship or an existing one? Was he nervous because he hadn't had sex for some time? Experts advise men may need to take up to 8 doses of a PDE5i on different occasions before they are considered a non-responder. In this case it may be worth advising Mr. W to try the product on his own to get used to the effects before proceeding to sexual intercourse. If after a few attempts the product is still not providing a satisfactory response, Mr. W should be advised to come back to speak to you or to visit his GP for further advice.

Case study 4

Mr. R should be taken through the Checklist to establish his symptoms and to look at the underlying issues. You know he is a smoker and there is a possibility that he is not in the best of health. The Checklist is designed to help you explore these issues and ascertain his suitability for the product. It is important to remember ED may not be the only concern; Mr. R has some signs that could indicate untreated depression. As such Mr. R should be advised to see his doctor to get a thorough assessment of his physical and mental health. The decision to supply the product will depend on the answers he gives you and this guide will help you with determining this for the wide range of men you may need to talk to.

7

Essential information

Name of product: VIAGRA CONNECT 50mg Film Coated Tablets
Active ingredient(s): Sildenafil
Product licence number: PL 00165/0392
Name and address of the product licence holder: Pfizer Consumer Healthcare Ltd, Ramsgate Road, Sandwich, Kent, CT13 9NJ, UK
Supply classification: P
Indications: For Erectile Dysfunction in adult men.

Side Effects: The safety profile of VIAGRA is based on > 9,000 patients in > 70 double-blind placebo controlled clinical studies. The most commonly reported adverse reactions in clinical studies among sildenafil treated patients were headache, flushing, dyspepsia, nasal congestion, dizziness, nausea, hot flush, visual disturbance, cyanopsia and vision blurred. Adverse reactions from post marketing surveillance has been gathered covering an estimated period >10 years. Because not all adverse reactions are reported to the Marketing Authorisation Holder and included in the safety database, the frequencies of these reactions cannot be reliably determined. Very Common (≥1/10): Headache. Common (≥1/100 and <1/10): Dizziness, Visual colour distortions (Chloropsia, Chromatopsia, Cyanopsia, Erythropsia and Xanthopsia), Visual disturbance, Vision blurred, Flushing, hot flush, nasal congestion, nausea, dyspepsia. Uncommon (≥1/1,000 and <1/100): Rhinitis, hypersensitivity; somnolence; hypoaesthesia, Lacrimation disorders (Dry eye, Lacrimal disorder and Lacrimation increased) Eye pain, Photophobia, Photopsia, Ocular hyperaemia, Visual brightness, Conjunctivitis, vertigo, tinnitus, tachycardia, palpitations, hypertension, hypotension, epistaxis, sinus congestion, Gastro oesophagael reflux disease, Vomiting, Abdominal pain upper, Dry mouth, rash, myalgia, pain in extremity, haematuria, chest pain, fatigue, feeling hot, heart rate increased. Rare (≥1/10,000 and <1/1,000): Cerebrovascular accident, Transient ischaemic attack, Seizure,* Seizure recurrence,* Syncope, Non arteritic anterior ischaemic optic neuropathy (NAION)*, Retinal vascular occlusion*, Retinal haemorrhage, Arteriosclerotic retinopathy, Retinal disorder, Glaucoma, Visual field defect, Diplopia, Visual acuity reduced, Myopia, Asthenopia, Vitreous floaters, Iris disorder, Mydriasis, Halo vision, Eye oedema, Eye swelling, Eye disorder, Conjunctival hyperaemia, Eye irritation, Abnormal sensation in eye, Eyelid oedema, Scleral discoloration, deafness, Sudden cardiac death*, Myocardial infarction, Ventricular arrhythmia*, Atrial fibrillation, Unstable angina, Throat tightness, Nasal oedema, Nasal dryness, Hypoaesthesia oral, Stevens Johnson Syndrome (SJS)*, Toxic Epidermal Necrolysis (TEN)* ,Penile haemorrhage, Priapism*, Haematospermia, Erection increased, Irritability

Precautions: Erectile dysfunction can be associated with a number of contributing conditions, e.g. hypertension, *diabetes mellitus*, hypercholesterolaemia or cardiovascular disease. As a result, all men with erectile dysfunction should be advised to consult their doctor within 6 months for a clinical review of potential underlying conditions and risk factors associated with erectile dysfunction (ED). If symptoms of ED have not improved after taking VIAGRA CONNECT on several consecutive occasions, or if their erectile dysfunction worsens, the patient should be advised to consult their doctor. Cardiovascular risk factors: Since there is a degree of cardiac risk associated with sexual activity, the cardiovascular status of men should be considered prior to initiation of therapy. Agents for the treatment of erectile dysfunction, including sildenafil, are not recommended to be used by those men who with light or moderate physical activity, such as walking briskly for 20 minutes or climbing 2 flights of stairs, feel very breathless or experience chest pain. The following patients are considered at low cardiovascular risk from sexual activity: patients who have been successfully revascularised (e.g. via coronary artery bypass grafting, stenting, or angioplasty), patients with asymptomatic controlled hypertension, and those with mild valvular disease. These patients may be suitable for treatment but should consult a doctor before resuming sexual activity. Patients previously diagnosed with the following must be advised to consult with their doctor before resuming sexual activity: uncontrolled hypertension, moderate to severe valvular disease, left ventricular dysfunction, hypertrophic obstructive and other cardiomyopathies, or significant arrhythmias. Sildenafil has vasodilator properties, resulting in mild and transient decreases in blood pressure (see section 5.1). Patients with increased susceptibility to vasodilators include those with left ventricular outflow obstruction (e.g., aortic stenosis), or those with the rare syndrome of multiple system atrophy manifesting as severely impaired autonomic control of blood pressure. Men with these conditions must not use the product without consulting a doctor. Sildenafil potentiates the hypotensive effect of nitrates (see section 4.3). Serious cardiovascular events, including myocardial infarction, unstable angina, sudden cardiac death, ventricular arrhythmia, cerebrovascular haemorrhage, transient ischaemic attack, hypertension and hypotension have been reported post-marketing in temporal association with the use of sildenafil. Most, but not all, of these patients had pre-existing cardiovascular risk factors. Many events were reported to occur during or shortly after sexual intercourse and a few were reported to occur shortly after the use of sildenafil without sexual activity. It is not possible to determine whether these events are related

directly to these factors or to other factors. Priapism: Patients who have conditions which may predispose them to priapism (such as sickle cell anaemia, multiple myeloma or leukaemia), should consult a doctor before using agents for the treatment of erectile dysfunction, including sildenafil. Prolonged erections and priapism have been occasionally reported with sildenafil in post-marketing experience. In the event of an erection that persists longer than 4 hours, the patient should seek immediate medical assistance. If priapism is not treated immediately, penile tissue damage and permanent loss of potency could result. Concomitant use with other treatments for erectile dysfunction: The safety and efficacy of combinations of sildenafil with other treatments for erectile dysfunction have not been studied. Therefore the use of such combinations is not recommended. Effects on vision: Cases of visual defects have been reported spontaneously in connection with the intake of sildenafil and other PDE5 inhibitors (see section 4.8). Cases of non-arteritic anterior ischaemic optic neuropathy, a rare condition, have been reported spontaneously and in an observational study in connection with the intake of sildenafil and other PDE5 inhibitors (see section 4.8). Patients should be advised that in the event of any sudden visual defect, they should stop taking VIAGRA CONNECT and consult a physician immediately (see section 4.3). Concomitant use with CYP3A4 inhibitors: Pharmacokinetic analysis of clinical trial data indicated a reduction in sildenafil clearance when co-administered with CYP3A4 inhibitors (such as ketoconazole, itraconazole, erythromycin, cimetidine). Although, no increased incidence of adverse events was observed in these patients, they should be advised to consult a doctor before taking VIAGRA CONNECT as a 25 mg tablet may be more suitable for them (see section 4.5 for further information). Concomitant use with alpha-blockers: Caution is advised when sildenafil is administered to patients taking an alpha-blocker, as the co-administration may lead to symptomatic hypotension in a few susceptible individuals (see section 4.5). This is most likely to occur within 4 hours post sildenafil dosing. In order to minimise the potential for developing postural hypotension, patients should be hemodynamically stable on alpha-blocker therapy prior to initiating sildenafil treatment. Thus, patients taking alpha blockers should be advised to consult their doctor before taking VIAGRA CONNECT as a 25 mg tablet may be more suitable for them. Treatment should be stopped if symptoms of postural hypotension occur, and patients should seek advice from their doctor on what to do. Effect on bleeding: Studies with human platelets indicate that sildenafil potentiates the antiaggregatory effect of sodium nitroprusside in vitro. There is no safety information on the administration of sildenafil to patients with bleeding disorders or active peptic ulceration. Therefore the use of sildenafil is not recommended in those patients with history of bleeding disorders or active peptic ulceration, and should only be administered after consultation with a doctor. Hepatic impairment: Patients with hepatic impairment must be advised to consult their doctor before taking VIAGRA CONNECT, since a 25 mg tablet may be more suitable for them (see section 4.2 and 5.2 for further information). Renal impairment: Patients with severe renal impairment (creatinine clearance <30 mL/min), must be advised to consult their doctor before taking VIAGRA CONNECT, since a 25 mg tablet may be more suitable for them (see section 4.2 and 5.2 for further information). Lactose: The film coating of the tablet contains lactose. VIAGRA CONNECT should not be administered to men with rare hereditary problems of galactose intolerance, Lapp lactase deficiency or glucose-galactose malabsorption. Use with alcohol: Drinking excessive alcohol can temporarily reduce a man's ability to get an erection. Men should be advised not to drink large amounts of alcohol before sexual activity.

Contra-indications: Hypersensitivity to the active substance or to any of the excipients listed in section 6.1. Consistent with its known effects on the nitric oxide/cyclic guanosine monophosphate (cGMP) pathway (see section 5.1), sildenafil was shown to potentiate the hypotensive effects of nitrates, and its co-administration with nitric oxide donors (such as amyl nitrite) or nitrates in any form is therefore contraindicated. Co-administration of VIAGRA CONNECT with ritonavir (a highly potent P450 enzyme inhibitor) is contraindicated (see section 4.5). The co-administration of phosphodiesterase type 5 (PDE5) inhibitors, including sildenafil, with guanylate cyclase stimulators, such as riociguat, is contraindicated as it may potentially lead to symptomatic hypotension (see section 4.5). Agents for the treatment of erectile dysfunction, including sildenafil, should not be used by those men for whom sexual activity may be inadvisable, and these patients should be referred to their doctor. This includes patients with severe cardiovascular disorders such as a recent (6 months) acute myocardial infarction (AMI) or stroke, unstable angina or severe cardiac failure. Sildenafil should not be used in patients with severe hepatic impairment, hypotension (blood pressure < 90/50 mmHg) and known hereditary degenerative retinal disorders such as *retinitis pigmentosa* (a minority of these patients have genetic disorders of retinal phosphodiesterases). This is because the safety of sildenafil has not been studied in these sub-groups of patients, and its use is therefore contraindicated. Sildenafil is contraindicated in patients who have loss of vision in one eye because of non-arteritic anterior ischaemic optic neuropathy (NAION), regardless of whether this episode was in connection or not with previous PDE5 inhibitor exposure (see section 4.4). VIAGRA CONNECT should not be used in patients with anatomical deformation of the penis (such as angulation, cavernosal fibrosis or Peyronie's disease). VIAGRA CONNECT is not indicated for use by women. The product is not intended for men without erectile dysfunction. This product is not intended for men under 18 years of age.

Dosage and Method of use: For Oral Use: Adults: The recommended dose is one 50 mg tablet taken with water approximately one hour before sexual activity. The maximum recommended dosing frequency is once per day. If VIAGRA CONNECT is taken with food, the onset of activity may be delayed compared to the fasted state (see section 5.2). Patients should be advised that they may need to take VIAGRA CONNECT a number of times on different occasions (a maximum of one 50 mg tablet per day), before they can achieve a penile erection satisfactory for sexual activity. If after several attempts on different dosing occasions patients are still not able to achieve a penile erection sufficient for satisfactory sexual activity, they should be advised to consult a doctor. Elderly: Dosage adjustments are not required in elderly patients (≥ 65 years old). Renal Impairment: No dosage adjustments are required for patients with mild to moderate renal impairment. However, since sildenafil clearance is reduced in individuals with severe renal impairment (creatinine clearance <30ml/min), individuals previously diagnosed with severe renal impairment must be advised to consult their doctor before taking VIAGRA CONNECT, since a 25 mg tablet may be more suitable for them (see section 4.4 for further information). Hepatic Impairment: Sildenafil clearance is reduced in individuals with hepatic impairment (e.g. cirrhosis). Individuals previously diagnosed with mild to moderate hepatic impairment must be advised to consult their doctor before taking VIAGRA CONNECT, since a 25 mg tablet may be more suitable for them (see section 4.4 for further information). The safety of sildenafil has not been studied in patients with severe hepatic impairment, and its use is therefore contraindicated (see section 4.3). Paediatric population: VIAGRA CONNECT is not indicated for individuals below 18 years of age. Use in patients taking other medicinal products: Pharmacokinetic analysis of clinical trial data indicated a reduction in sildenafil clearance when co-administered with CYP3A4 inhibitors (such as ritonavir, ketoconazole, itraconazole, erythromycin, cimetidine). With the exception of ritonavir, for which co-administration with sildenafil is contraindicated (see section 4.3), individuals receiving concomitant treatment with CYP3A4 inhibitors must be advised to consult their doctor before taking VIAGRA CONNECT, since a 25 mg tablet may be more suitable for them (see section 4.4 for further information). In order to minimise the potential of developing postural hypotension in patients receiving alpha blocker treatment (e.g. alfuzosin, doxazosin or tamsulosin), patients should be stabilised on alpha blocker therapy prior to initiating sildenafil treatment. Thus, patients taking alpha blockers must be advised to consult their doctor before taking VIAGRA CONNECT since a 25 mg tablet may be more suitable for them (see sections 4.4 and 4.5).

Cost: RRP excluding VAT; 4 Pack £16.66 and 8 Pack £29.16

Date: November 2017

REFERENCES

References

1. Lue T, *et al.* Summary of the recommendations on sexual dysfunctions in men. *J Sex Med* 2004;1:6–23.
2. NICE. Clinical knowledge summaries. Erectile dysfunction. Available at: http://cks.nice.org.uk/erectile-dysfunction. Accessed Nov 2017.
3. Miner M. Erectile dysfunction: a harbinger or consequence: does its detection lead to a window of curability? *J Androl* 2011;32:125–134.
4. The British Association of Urological Surgeons. Erectile dysfunction. Frequently asked questions. Available at: **https://www.baus.org.uk/patients/conditions/3/erectile_dysfunction_impotence**. Accessed Nov 2017.
5. Rosen R. Psychogenic erectile dysfunction: classification and management. *Urol Clin North Am* 2001;28:269–278.
6. Conaglen H, *et al.* Drug-induced sexual dysfunction in men and women. *Aust Prescr* 2013;36:42–52.
7. Nunes KP, *et al.* New insights into hypertension-associated erectile dysfunction. *Curr Opin Nephrol Hypertens* 2012;21:163–170.
8. Fusco F, *et al.* The impact of non-urologic drugs on sexual function in men. *Arch Ital Urol Androl* 2014;86:1.
9. Lee JC. Club drugs' and erectile function: far from sexual ecstasy. *J Sex Reprod Med* 2002;2:28–30.
10. Lasker GF, *et al*. A review of the pathophysiology and novel treatments for erectile dysfunction. *Adv Pharmacol Sci* 2010;2010:1–10.
11. WebMD. Drugs linked to erectile dysfunction. Available at: **http://www.webmd.com/erectile-dysfunction/guide/drugs-linked-erectile-dysfunction**. Accessed Jun 2017.
12. Muneer A, *et al.* Erectile dysfunction. *BMJ* 2014;348:g129.
13. Cappelleri JC, *et al.* Clinically meaningful improvement on the self-esteem and relationship questionnaire in men with erectile dysfunction. *Qual Life Res* 2007;16:1203–1210.
14. Ballard SA, *et al.* Effects of sildenafil on the relaxation of human corpus cavernosum tissue *in vitro* and on the activities of cyclic nucleotide phosphodiesterase isozymes. *J Urol* 1998:159:2164–2171.
15. VIAGRA CONNECT 50 mg film-coated tablets (sildenafil): Summary of Product Characteristics. Pfizer Consumer Healthcare Ltd.
16. British Society for Sexual Medicine. Guidelines on the management of erectile dysfunction, 2013. Available at: **http://bssmorguk.ipage.com/wp-content/uploads/2017/05/BSSM_ED_Management_Guidelines_2013.pdf**. Accessed Jun 2017.
17. Nehra A, *et al.* The Princeton III consensus recommendations for the management of erectile dysfunction and cardiovascular disease. *Mayo Clin Proc* 2012;87:766–778.

Notes

Notes

Pfizer Consumer Healthcare

Date of preparation: November 2017 | PP-PHE-GBR-0021
Printed January 2018

Online Resource 2 Flowchart of respondents through the study.

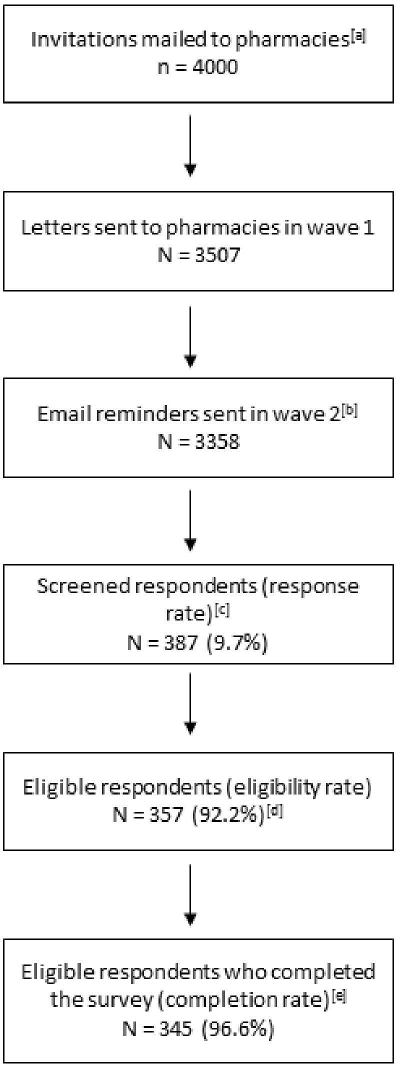

[a]There were 3 instances in which more than 1 pharmacist was invited from a single pharmacy (identified by their unique respondent code)

[b]A total of 6865 emails were sent in 2 separate waves. If a single pharmacy received a letter and an email, the first email counted as a reminder. The second wave excluded respondents and nonviable emails

[c]All respondents who accessed the survey using their unique code (regardless of whether questions were answered or not); response rate calculated as a percentage of those invited

[d]Eligibility rate calculated as a percentage of screened respondents

[e]Respondents who completed the survey, calculated as a percentage of eligible respondents

Online Resource 3 Pharmacist participation in the Viagra Connect Training Guide to Pharmacists

	Pharmacists (n = 345) n (%)
Participated in the Viagra Connect pharmacist training	
Yes	238 (69.0)
No	60 (17.4)
Don't recall	47 (13.6)
Main reason for not accessing the training[a,b]	
Did not know about it	35 (32.7)
Other	26 (24.3)
PGD-trained (or equivalent) for PDE5I	21 (19.6)
Did not have time	19 (17.8)
Had difficulty accessing materials electronically	6 (5.6)
Format of Viagra Connect pharmacist training[c,d]	
Printed training materials in the mail	129 (54.2)
Online training provided by pharmacist's company	86 (36.1)
Online training via the Viagra Connect website	67 (28.2)
During a regional pharmacist meeting	53 (22.3)
During face-to-face training in their pharmacy	44 (18.5)
Online training through a website accredited by the RPS	40 (16.8)
Other	4 (1.7)

PDE5I phosphodiesterase type-5 inhibitor, *PGD* patient group direction, *RPS* Royal Pharmaceutical Society

[a]Percentages calculated based on the sample presented with this question because of skip logic (those who answered "No" and "Don't recall"; n = 107)

[b]Only a single answer could be chosen

[c]Percentages calculated based on the sample presented with this question because of skip logic (those who answered "Yes"; n = 238)

[d]Percentages may not total 100% as more than 1 response could be selected

Online Resource 4 Pharmacist knowledge of key risk messages pertaining to Viagra Connect and other behind-the-counter products

Main reference used for the safe dispensing practices of Viagra Connect	Pharmacists (n = 345)<br>n (%)
The Viagra Connect pharmacist training *Essential Information for the Supply of Viagra Connect*	161 (46.7)
Viagra Connect Pharmacy Checklist	115 (33.3)
The Viagra Connect SmPC	52 (15.1)
Own pharmacy's medication reference (e.g. BNF)	8 (2.3)
Viagra Connect website	5 (1.4)
The Viagra Connect box label	2 (0.6)
Pharmacy journals	1 (0.3)
Sponsor sales professional	1 (0.3)

BNF British National Formulary, *SmPC* summary of product characteristics

Online Appendix Questions asked in the survey

**Question**	**Possible Responses**
Question 1: Do you agree to participate in this survey?	Yes<br>No<br>Discontinued
Question 2: Are you a practising pharmacist in the UK?	Yes<br>No<br>Question not asked<br>Discontinued
Question 3: Have you received at least 1 patient face-to-face request for Viagra Connect in the past 6 months?	Yes<br>No<br>Question not asked<br>Discontinued
Question 4: Are you or any of your immediate family members currently employed by Pfizer, another pharmaceutical company, United BioSource Corporation (UBC) or contracted by regulatory bodies [eg, European Medicines Agency (EMA) or Medicines and Healthcare Products Regulatory Agency (MHRA)]?	Yes<br>No<br>Question not asked<br>Discontinued
Question 5: Which best describe the type of pharmacy setting you primarily work in? Select ONE option.	Face-to-face<br>Face-to-face and online<br>Online only<br>Question not asked<br>Discontinued
Question 6: How many patients do you estimate have requested to purchase Viagra Connect from you in the past 6 months?	1–2<br>3–5<br>6–10<br>11–20<br>21–30<br>More than 31<br>I don't know
Question 7: Of those patients who requested to purchase Viagra Connect from you, what percentage do you estimate were not suitable to be supplied the product?	Up to 10%<br>11–50%<br>51–90%<br>More than 90%<br>I don't know
Question 8: Which best describes the place of your pharmacy?	Urban (city, large town or suburban area)<br>Rural (small town, village, countryside)

Question 9: What type of pharmacy setting do you primarily work in? Select ONE option.	Large multiple-pharmacy chain (more than 100 stores)<br>Small multiple-pharmacy chain (6 to 99 stores)<br>Independent Pharmacy (1 to 5 stores)
Question 10: How long have you been dispensing medications as a pharmacist?	Less than 2 years<br>2–5 years<br>6–10 years<br>11–20 years<br>Greater than 20 years
Question 11: Which best represents your age group?	22–30 years<br>31–40 years<br>41–60 years<br>More than 61 years old
Question 12: Which gender are you?	Male<br>Female<br>Prefer not to answer
Question 13: Please answer True/False/Don't know for the following statements:	True<br>False<br>Don't know
Viagra Connect is only intended for use by men over 45 years of age who are experiencing Erectile Dysfunction (ED).	
Viagra Connect must not be supplied to men who do not have Erectile Dysfunction (ED).	
Men currently prescribed 50 mg sildenafil can be supplied Viagra Connect if a) they meet the criteria for pharmacy supply and b) they do not take more than 50 mg daily.	
Men who are using a different dose of sildenafil or another Erectile Dysfunction (ED) treatment can also be supplied with Viagra Connect.	
Question 14: The following patients should NOT be supplied with Viagra Connect but should be referred to their doctor.	True<br>False<br>Don't know
Those who have been advised by their doctor that they are not fit enough for physical or sexual activity.	
Those who experience breathlessness or chest pain with light or moderate physical activity (eg	

walking briskly for 20 mins or climbing 2 flights of stairs).	
Those who have had a heart attack or stroke more than 6 months ago.	
Question 15: A patient with which of the following cardiovascular conditions MAY be supplied with Viagra Connect?	Yes<br>No<br>Don't know
Low blood pressure	
Uncontrolled hypertension	
Controlled hypertension	
Unstable angina	
Arrhythmia	
Valvular heart disease	
Cardiomyopathy	
Left Ventricular outflow obstruction or aortic narrowing	
Severe cardiac failure	
Question 16: Look at the list of drugs below and select whether or not they can be used with Viagra Connect.	Yes<br>No<br>Don't know
Nitrates for chest pain	
Riociguat for lung problems	
Ritonavir for HIV infection	
Omeprazole or other proton pump inhibitors (PPIs)	
CYP3A4 inhibitors (eg, saquinavir, cimetidine, itraconazole, ketoconazole, erythromycin or rifampicin, diltiazem)	
Alpha-blockers (eg, alfuzosin, doxazosin or tamsulosin)	
Beta-blockers	
Recreational drugs called "poppers" (eg, amyl nitrite)	
Question 17: Patients with the following concomitant diseases should NOT be supplied with Viagra Connect but should be referred to their doctor.	True<br>False<br>Don't know
Hepatic disease	
Severe renal impairment	
Osteoarthritis or other musculoskeletal disorders	

Loss of vision due to optic nerve damage or inherited eye disease (such as retinitis pigmentosa)	
Question 18: Which of the following MAY be a cause of Erectile Dysfunction (ED) and should be considered when assessing a patient for suitability for Viagra Connect.	True<br>False<br>Don't know
Undiagnosed depression	
Anxiety	
Excessive alcohol use	
Hypertension	
Diabetes mellitus	
Hypercholesterolemia	
Cardiovascular disease	
Osteoarthritis	
Question 19: If a patient requests Viagra Connect but shows evidence of undiagnosed depression, anxiety or excessive alcohol use, which action should you take? Please select True/False/Don't Know.	True<br>False<br>Don't know
Provide lifestyle advice	
Recommend follow-up with his doctor	
Question 20: Please select True/False/Don't Know for the following statements.	True<br>False<br>Don't know
Pharmacists should provide lifestyle advice to all men with Erectile Dysfunction (ED) who request Viagra Connect.	
Lifestyle advice includes losing weight, stopping smoking, cutting back on alcohol and recreational drugs, exercising regularly and reducing stress.	
All patients supplied with Viagra Connect should be advised to consult their doctor within 6 months from the first supply.	
All patients who are refused Viagra Connect should be advised to contact their doctor.	
Question 21: Patients should be advised to STOP TAKING Viagra Connect and seek medical attention	True<br>False<br>Don't know

immediately if they experience the following symptoms.	
Chest pains	
Persistent and sometimes painful erection lasting longer than 4 hours	
Dyspepsia	
Sudden decrease or loss of vision	
Headache	
Allergic reaction (with symptoms of wheeziness, difficulty breathing or dizziness)	
Nausea	
Serious skin reactions (eg, Stevens-Johnson Syndrome [SJS])	
Seizures or fits	
Question 22: Which of the following sources is your main reference about the safe dispensing practices of Viagra Connect?	Please select ONE only:
The Viagra Connect Summary of Product Characteristics (SmPC)	
The Viagra Connect Box Label	
The Viagra Connect Pharmacist Training *Essential Information for the Supply of Viagra Connect*	
The Viagra Connect Pharmacist Checklist	
My pharmacy's medication reference eg, British National Formulary (BNF)	
Viagra Connect website	
Pharmacy journals (eg, The Pharmaceutical Journal, Pharmacy, The Clinical Pharmacist)	
Colleagues	
Pfizer sales professional	
Other	
Question 23: Regarding the counselling of patients seeking to purchase Viagra Connect:	Yes<br>No
I am comfortable counselling the patient each time they request Viagra Connect	
My experience is that patients are interested in learning and open to discussing their health	

Patients often tell me that they do not want to go through counselling	
If I have covered some of the issues with the patient during a previous purchase I am less likely to	
I think that these discussions (ie, Viagra Connect supply counselling) make some patients uncomfortable<br>and unwilling to divulge information	
The counselling about Viagra Connect makes me uncomfortable	
It is difficult to counsel patients because there is no private space to do so in my pharmacy	
Patients have expressed dissatisfaction with counselling	
Question 24: Do you use the optional *Viagra Connect Pharmacy Checklist* at the point of supply?	Yes<br>No<br>I do not recall
Question 25: From the list below please select reason(s) for NOT utilising the optional *Viagra Connect Pharmacy Checklist*.	Select ALL that apply:
I was not aware of the checklist	
I used it for a while after the training but did not think I needed to continue to use it as it was no longer required as a prompt.	
I am PGD (patient group direction)-trained (or equivalent) for PDE-5i's	
I am familiar with the information and I do not think I need the checklist to consult the patient	
I use it only with new patients but not with returning patients	
Other	
N/A (Answered "Yes" to Question 24)	
Question 26: How frequently do you use the optional *Viagra Connect Pharmacy Checklist*?	All the time for all customers presenting<br>Only used after first trained and no longer required as a prompt<br>Never<br>N/A (Answered "No" or "I do not recall" to Question 24)

For new requests for Viagra Connect[	
For patients returning for repeat supplies of Viagra Connect	
Question 27: How frequently do you provide the patient with the tear-off slip contained on the bottom of the checklist?	Always<br>Sometimes<br>Never
If I have supplied Viagra Connect	
If I have denied the patient's request for Viagra Connect	
Question 28: Did you participate in the Viagra Connect pharmacist training?	Yes<br>No<br>I don't recall
Question 29: What was your main reason for NOT accessing the training?	Select ONE only.
I did not know about it	
I have not had time	
I had difficulty accessing the materials electronically	
I am PGD (patient group direction)-trained (or equivalent) for phosphodiesterase type 5 inhibitors (PDE-5i's)	
Other	
N/A (Answered "Yes" to Question 28)	
Question 30: What format of the Viagra Connect pharmacist training did you participate in?	Select ALL that apply
Online training through a website for pharmacists accredited by the Royal Pharmaceutical Society (RPS)	
During a regional pharmacist meeting	
Online training via the Viagra Connect Website	
Online training provided by company eg, Boots own training	
I received printed training materials in the mail	
During a face-to-face training in my pharmacy	
Other	
N/A (Answered "No" or "I don't recall" to Question 28)	

Question 31: Did you read the *Essential Information for the Supply of Viagra Connect* guide for pharmacists?	Yes<br>No
Question 32: Before today, were you aware of the *Viagra Connect Pharmacy Checklist?*	Yes<br>No
Question 33: Did you find the Viagra Connect pharmacist training/reference tools to be of use in your practice? Please select only ONE response for each tool listed below:	Not useful<br>Somewhat useful<br>No opinion/not sure<br>Very useful<br>Extremely useful<br>Not applicable
Viagra Connect Training Guide to Pharmacists	
Viagra Connect Pharmacist Checklist	
Tear-off slip at the bottom of the pharmacist checklist	
